# Recognition motif and mechanism of ripening inhibitory peptides in plant hormone receptor ETR1

**DOI:** 10.1038/s41598-018-21952-3

**Published:** 2018-03-01

**Authors:** Dalibor Milić, Markus Dick, Daniel Mulnaes, Christopher Pfleger, Anna Kinnen, Holger Gohlke, Georg Groth

**Affiliations:** 1 Institute of Biochemical Plant Physiology and Bioeconomy Science Center (BioSC), Heinrich Heine University Düsseldorf, Düsseldorf, Germany; 2 Institute of Pharmaceutical and Medicinal Chemistry and Bioeconomy Science Center (BioSC), Heinrich Heine University Düsseldorf, Düsseldorf, Germany; 3 John von Neumann Institute for Computing (NIC), Jülich Supercomputing Centre (JSC) & Institute for Complex Systems - Structural Biochemistry (ICS 6), Forschungszentrum Jülich GmbH, Jülich, Germany; 4 Present Address: Department of Structural and Computational Biology, Max F. Perutz Laboratories, University of Vienna, Vienna Biocenter, Vienna, Austria; 5 Present Address: Division of Chemistry and Chemical Engineering, California Institute of Technology, Pasadena, California, USA

## Abstract

Synthetic peptides derived from ethylene-insensitive protein 2 (EIN2), a central regulator of ethylene signalling, were recently shown to delay fruit ripening by interrupting protein–protein interactions in the ethylene signalling pathway. Here, we show that the inhibitory peptide NOP-1 binds to the GAF domain of ETR1 – the prototype of the plant ethylene receptor family. Site-directed mutagenesis and computational studies reveal the peptide interaction site and a plausible molecular mechanism for the ripening inhibition.

## Introduction

**Figure 1:**
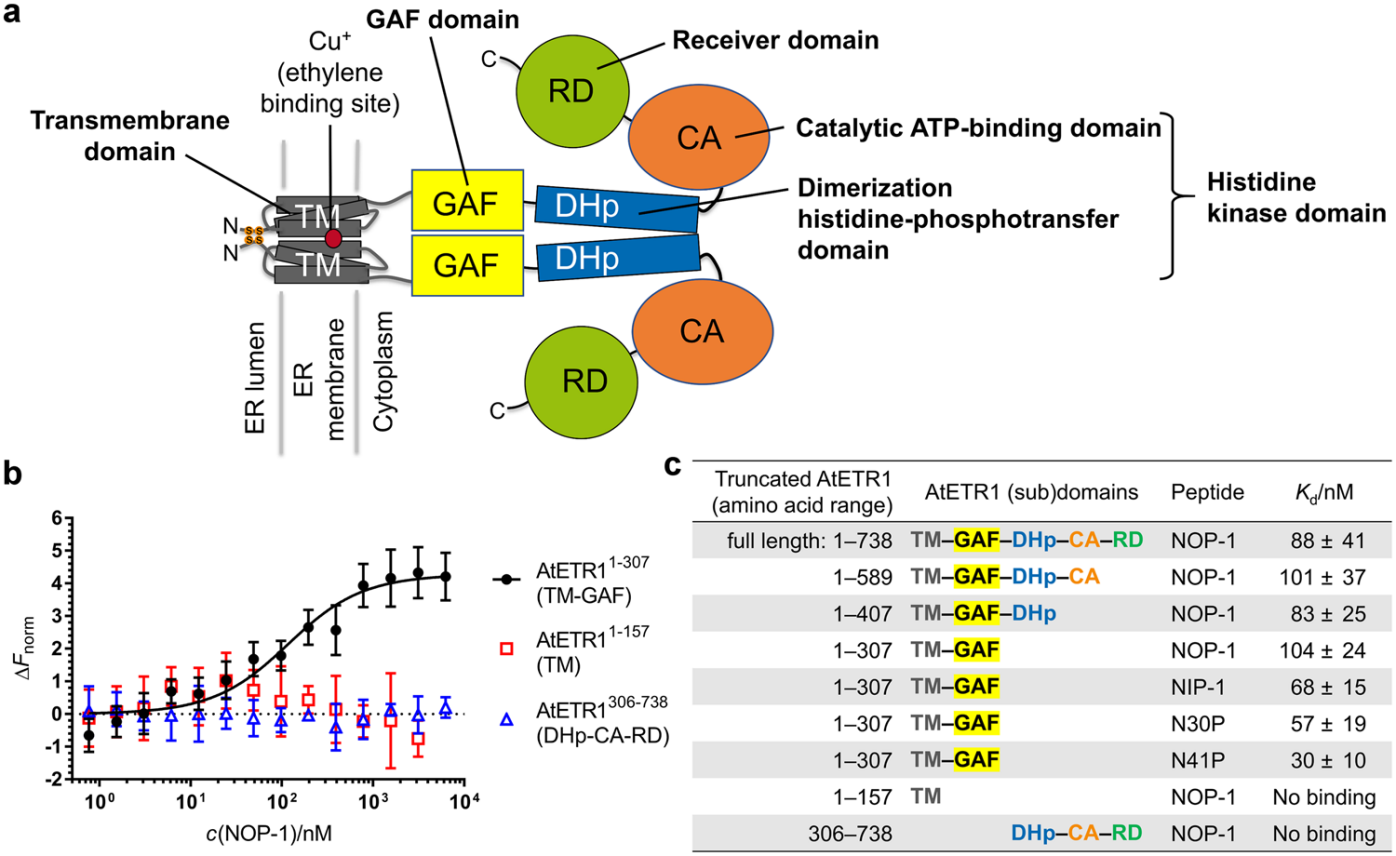
Identification of the AtETR1 domain interacting with inhibitory octapeptide NOP-1. (**a**) Modular organization of the AtETR1 structure. The receptor forms a covalent dimer *via* two disulfide bridges at the N-terminus. The ethylene binding site (Cu^+^ ion) is situated at the interface of two α-helical transmembrane (TM) domains immersed in a membrane of the endoplasmic reticulum (ER). The highly flexible cytoplasmic part of AtETR1 is composed of four domains: a GAF, a dimerization histidine-phosphotransfer (DHp), a catalytic ATP-binding (CA), and a receiver (RD) domain. DHp and CA domains are parts of a histidine kinase functional unit. (**b**) Binding of NOP-1 to the truncated AtETR1 constructs studied by microscale thermophoresis (MST). ∆*F*_norm_ is a relative normalized fluorescence measured for a fluorescently labelled protein at constant concentration (25 nM) in the presence of NOP-1 at different concentrations, *c*(NOP-1). AtETR1^1–307^ (TM–GAF) still binds NOP-1, while AtETR1^1–157^ (TM) and AtETR1^306–738^ (DHp–CA–RD) show no binding. Mean values and standard deviations of ∆*F*_norm_ are plotted. (**c**) Dissociation constants (*K*_d_) determined in MST binding experiments with the truncated AtETR1 constructs. All corresponding binding curves are presented in Supplementary Fig. S2.

Ripening of climacteric fruits, such as apples and tomatoes, is induced by the plant hormone ethylene. Such fruits and vegetables are usually harvested, transported, and stored in a green, unripe state, and full ripening is then induced by ethylene exposure at the final destination shortly before delivery. In order to avoid fruit damage and spoilage due to overripening, strategies have been developed to control ripening and minimize postharvest losses^1^ by interfering with ethylene biosynthesis or signalling. Much of the current knowledge on signal perception and transduction of the plant hormone has been established by physiological, biochemical and genetic studies in the model plant *Arabidopsis thaliana*. Overall, more than a dozen genes have been implicated in the ethylene-signaling pathway, and their multi-stage interconnecting network has been tentatively determined using a combination of genetic and molecular approaches. In *Arabidopsis*, the ethylene signal is perceived by a family of five receptor proteins, which form homo- and heterodimers at the membrane of endoplasmic reticulum (ER) and function as negative regulators of the ethylene response^2–7^. The receptors are modular (Fig. 1a), organized similar to bacterial sensor histidine kinases and contain N-terminal transmembrane sensor domains (TM) followed by a cytosolic GAF domain (GAF), a dimerization histidine-phosphotransfer (DHp) and a catalytic ATP-binding (CA) domain forming the catalytic core, and a C-terminal response regulator domain (RD; not present in all members of the ethylene receptor family)^8,9^. Although the exact output of the receptors is still obscure, genetic studies demonstrate that in the absence of ethylene, receptors activate the Raf-like protein kinase CONSTITUTIVE TRIPLE RESPONSE 1 (CTR1), a negative regulator of the pathway^10^. Although CTR1 lacks any predicted transmembrane domains, it also resides at the ER membrane due to its physical interaction with the receptors^11^. Interaction with the receptors is considered critical for the induction of CTR1 kinase activity. Downstream of the receptors and the ER associated CTR1 kinase the membrane protein ETHYLENE INSENSITIVE 2 (EIN2) implements a positive regulatory role on ethylene signaling. The integral membrane protein was identified as the most crucial step in ethylene signaling since *ein2* is the only gene whose loss-of-function mutation confers complete ethylene insensitivity to the plant^12^. Recently, we identified inhibitory oligopeptides that delay ripening of tomatoes (*Solanum lycopersicum*) when applied onto the surface of an unripe fruit before or after its harvesting^13–15^. Their amino acid sequences are based on a highly conserved nuclear localization signal (NLS) found at the C-terminus of EIN2^16^. Molecular and genetic studies revealed that the C-terminal cytoplasmic part of EIN2 (EIN2-CEND) gets cleaved in the presence of ethylene by a so far unknown mechanism and has a crucial role in regulating expression of ethylene response genes^17–21^. Recent work in our laboratory showed that the synthetic inhibitory peptides derived from the NLS motif at the EIN2 C-terminus bind directly to ethylene receptors^14,15^ and disrupt their interactions with EIN2-CEND^13,14^.

In this report, we demonstrate that the inhibitory peptides bind to the GAF domain of ethylene receptor 1 (ETR1). Furthermore, the results of our experimental and computational biophysical studies not only indicate the peptide interaction site but also suggest a probable molecular mechanism of the ripening inhibition.

## Results and Discussion

To understand the structural basis of interactions between ethylene receptors and inhibitory peptides, we heterologously expressed and purified C-terminally truncated constructs of ETR1 from the plant model organism *A*. *thaliana* (AtETR1), which were successively lacking protein domain modules starting from the C-terminus (Fig. 1a and Supplementary Fig. S1). Our goal was to identify AtETR1 domain(s) crucial for the interaction with the archetypal inhibitory octapeptide NOP-1 (LKRYKRRL-NH_2_)^13–15^, the sequence of which matches exactly the NLS sequence found in EIN2 of most plant species^14^, including *A*. *thaliana* and tomato. Therefore, we used microscale thermophoresis to characterize binding of NOP-1 to the fluorescently labelled full-length AtETR1 and each of its four C-terminally truncated constructs (Fig. 1b,c and Supplementary Fig. S2). Out of these, AtETR1^1–157^, containing the transmembrane (TM) domain only, showed no binding to the inhibitory peptide. All other C-terminally truncated constructs bound NOP-1 with binding affinities very similar to those of the full-length protein (dissociation constant *K*_d_ = 88 ± 41 nM; Fig. 1c). To further explore the role of the histidine kinase (DHp and CA) or receiver domains (RD) in binding of NOP-1, we prepared AtETR1^306–738^ containing only these domains. To our surprise, we observed no binding of NOP-1 to AtETR1^306–738^ (Fig. 1b), thus ruling out our initial hypothesis that the NOP-1 binding site corresponds to a canonical phosphorylation site in the ETR1 histidine kinase or receiver domain^13^. Taken together, these results pinpointed the GAF domain as the ETR1 structural unit that interacts with NOP-1. Moreover, the three extended peptides NIP-1 (AFPKGKENLASVLKRYKRRL-NH_2_)^13^, N30P (GRTGTAAGDVAFPKGKENLASVLKRYKRRL-NH_2_), and N41P (KDVEMAISSRKGRTGTAAGDVAFPKGKENLASVLKRYKRRL-NH_2_) – all of which were derived from the AtEIN2 sequence and contain the NLS motif with additional 12, 22, or 33 upstream amino acid residues, respectively – also showed binding to AtETR1^1–307^ (Fig. 1c and Supplementary Fig. S2). Their binding affinities improved with increasing peptide length, highlighting the importance of the NLS-core motif in this interaction along with the positive correlation of sequence length on folding and/or stability of the biologicals (Supplementary Fig. S3).

Previous *in vivo* studies by various labs^7,22,23^ have demonstrated a crucial role of the GAF domain for noncovalent homo- and hetero-oligomerization of ethylene receptors. Even before these discoveries, several researchers proposed that non-covalent interactions between the receptors and formation of higher-order oligomers might have functional implications in ethylene signalling and could explain the high sensitivity and broad concentration range of ethylene response^24–27^.

To further understand the nature of peptide–GAF domain interactions, we first focused on predicting possible common structural motifs of peptides NOP-1, NIP-1, N30P, and N41P. We used 50 μs long molecular dynamics (MD) simulations, with three independent replicates for each system, in implicit solvent to perform *ab initio* folding simulations, motivated by recent successful studies^28,29^. In neither case did we see tertiary structure formation, and, except for specific regions (amino acids 7–9, 11–15 that tend to form α-helices), the major secondary structural elements were random coils (Supplementary Fig. S3a); these predictions were confirmed by CD spectroscopy (Supplementary Fig. S3b,c). Hence, it was not possible to identify a common structural motif. Nevertheless, such a result is not completely unexpected, considering the short length and high number of positive charges of the peptides, and the fact that the peptide sequences are part of the C-terminal domain of AtEIN2, which is predicted to be mainly disordered (60% disordered regions according to DISOPRED^30^).

**Figure 2:**
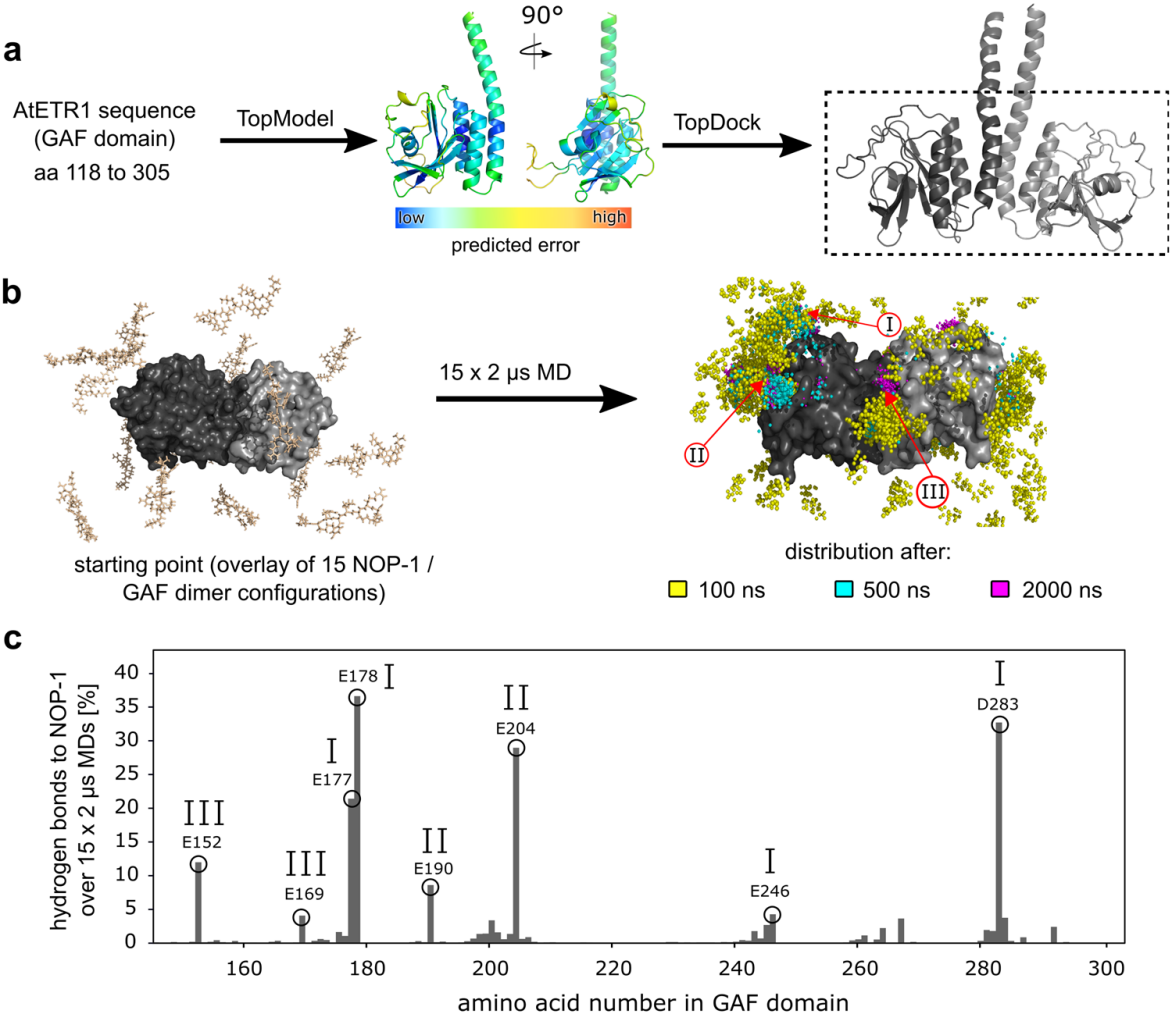
Molecular modelling of NOP-1 interactions with the GAF domain of AtETR1. (**a**) Model building of the GAF domain (dimeric form). Amino acids 118 to 305 of AtETR1 were used as a target sequence to build a homology model using TopModel^31^. The colouring of the monomeric structures represents the residue-wise uncertainty of the predicted model computed by TopScore. Next, protein–protein docking guided by positional restraints was performed to determine the interface between both monomeric subunits. As is known from experimental data (see Fig. 1b), amino acids 118 to 141 do not interact with NOP-1 and are not needed for the dimer formation. Thus, only the part of the protein shown in the dashed black box was used for further studies. (**b**) Starting from different initial NOP-1 positions (left, NOP-1 structures are coloured in beige, while the GAF domains are labelled in dark and light grey) 15 MD simulations of 2 µs length were performed. The cumulative distribution of the peptide after 100 ns (yellow), 500 ns (turquois), and 2000 ns (pink) over the 15 MD simulations is shown as points (representing the centre of mass of NOP-1) superimposed onto the average structure of the GAF dimer. The three main binding sites are highlighted by red arrows and labelled with Roman numerals (I to III). (**c**) The overall percentage of hydrogen bond and salt bridge formation with NOP-1 is shown for each residue of the GAF domain over the 15 MD simulations; results obtained for either domain in the GAF dimer were averaged. All residues chosen for mutation to alanine are labelled. The Roman numerals represent the corresponding binding sites as in panel b.

As no experimental structure of the ETR1 GAF domain has been reported so far, we used our in-house software package TopModel^31^ to build a structural model based on available templates (Supplementary Fig. S4 and Supplementary Table S1) applying the sequence of AtETR1118–305 as the target (PDB ID and chain identifier of the templates given, with sequence identity indicated in parentheses: 3P01_A (18%), 3TRC_A (15%), 3CI6_A (13%), 3W2Z_A (12%), and 1YKD_B (15%)). A structural alignment between the GAF domain model and the templates used is shown in Supplementary Fig. S5. The final model built by TopModel (Fig. 2a) was assessed with our in-house model quality assessment program TopScore (D. Mulnaes, H. Gohlke, unpublished results; see Materials and Methods section for details) to be 71% correct, with the majority of inaccuracies being located in the flexible loop regions (residues in AtETR1 228–247 and 257–272: 47% and 52% inaccuracies, respectively).

Previous findings suggest that ethylene receptors form a dimer in their simplest functional state that is also mediated by their GAF domains^32^. We therefore built a dimer model of the AtETR1 GAF domain using our in-house protein–protein docking software TopDock (D. Mulnaes, H. Gohlke, unpublished results). TopDock predicts protein–protein contacts based on a structure-based homology search that is independent of sequence. TopDock identified five different homologous interfaces (PDB ID and chain identifiers given: 3G6O_AB, 3IBJ_AB, 3K2N_AB, 3P01_AB, and 3TRC_AB) all of which indicate that the dimer interface consists of the N- and C-terminal helices of the GAF domain (Supplementary Fig. S6). TopDock-predicted residue–residue contacts from each homologous interface were used for restrained docking of the GAF domains with HADDOCK^33^. The docking solutions were pooled and clustered by TopDock, and ranked according to HADDOCK energy, cluster size, distance to cluster centroid, and fulfilment of predicted contacts to select a docking solution (Fig. 2a). Each monomeric subunit of our final model contains a central, antiparallel, seven-fold β-sheet, flanked by one short α-helix (amino acids 213–220) and three, parallel-oriented α-helices that cover the N- and C-terminal regions (amino acids 118–173 and 290–305). Both N-terminal α-helices form the dimeric interface resulting in a six-helix bundle in the homodimeric structure (Fig. 2a). MD simulations of the protein of 500 ns length in the absence of any peptide ligand revealed overall moderate structural variations within both monomers (Supplementary Fig. S7), when the unstructured loop regions (residues 222–290) were omitted.

To identify interaction sites on the GAF dimer to which NOP-1 binds, we performed 15 independent MD simulations of 2 µs length each of free NOP-1 diffusion around the dimer, motivated by our own experience^31^ and that of others^34,35^ in related studies. To prevent any bias, NOP-1 was randomly placed in the simulation box also containing the ETR1 GAF dimer and explicit solvent (Fig. 2b). Over the simulation times, the locations of NOP-1 at the GAF dimer converge to three binding regions (Fig. 2b): (I) in the upper loop region (residues 283–286), (II) nearby the central β-sheets (residues 190–205), and (III) at the helices of the dimeric interface (residues 152–170). The propensity of hydrogen bond and salt bridge formation between a protein residue and NOP-1, averaged over the entire MD simulation data, confirmed preferred NOP-1/GAF dimer interactions with the three sites (Fig. 2c).

**Figure 3:**
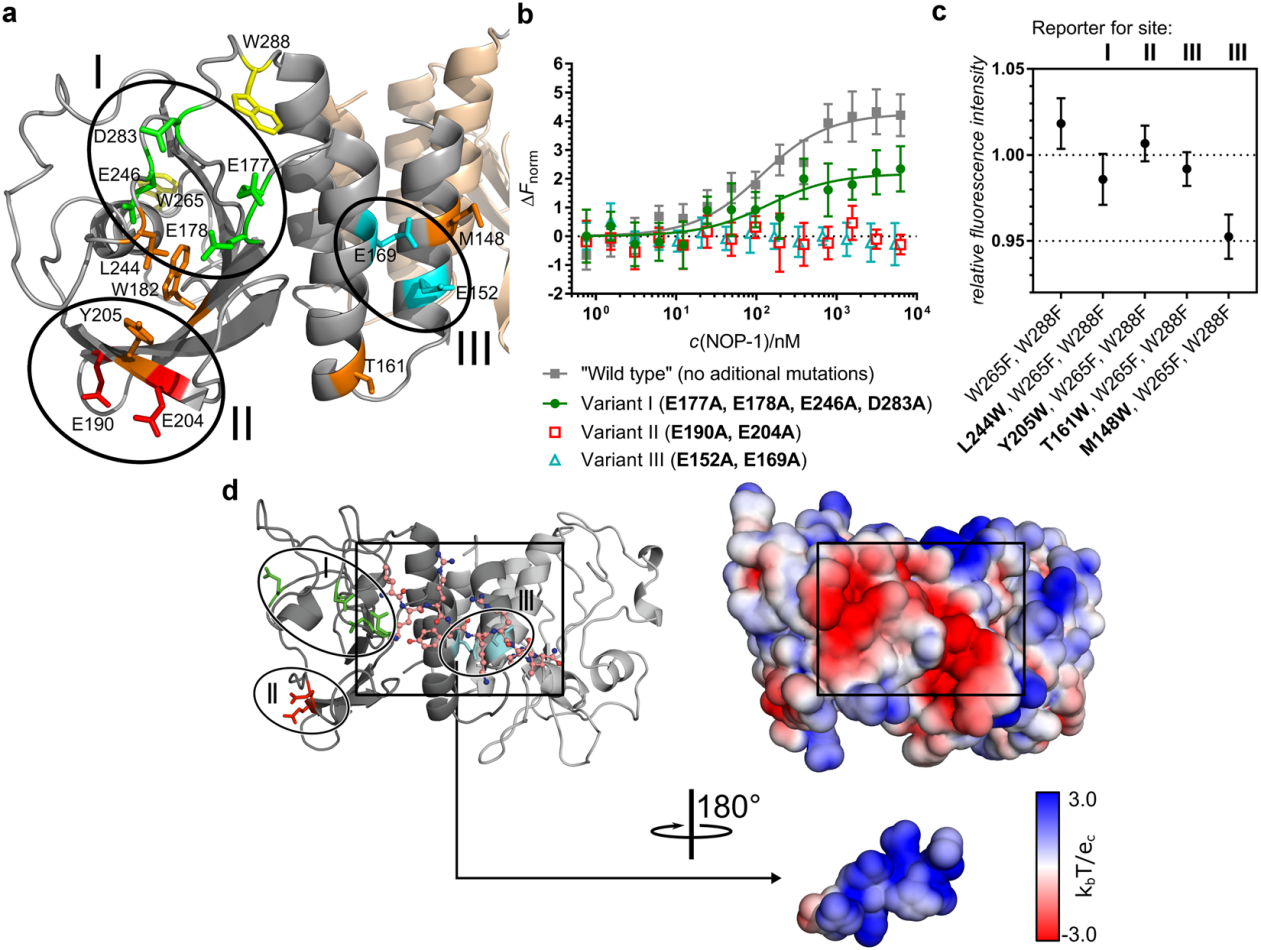
Evaluation of the predicted binding regions in the GAF domain of AtETR1. (**a**) Model of AtETR1 GAF domain with the highlighted acidic residues potentially involved in binding of NOP-1 (region I – *red*, region II – *green*, region III - *cyan*). Two tryptophans (W265 and W288) mutated to phenylalanine for the intrinsic fluorescence quenching experiments are shown in *yellow*. The remaining tryptophan (W182) and the four residues separately exchanged for tryptophan (fluorescence reporter) are highlighted in *orange*. (**b**) Binding of NOP-1 to the fluorescently labelled AtETR1^1–307^ and its three variants monitored via microscale thermophoresis (relative normalized fluorescence, ∆*F*_norm_). (**c**) Tryptophan fluorescence quenching of the AtETR1^1–307^ Trp-variants by NOP-1. Fluorescence intensity of each Trp-variant in the presence of 10-fold excess of NOP-1 is given relative to fluorescence intensity of each protein measured without NOP-1. The complete titration data are presented in Supplementary Fig. S8. Mean values and standard deviations of independent triplicate measurements are shown in panels (**b**) and (**c**). (**d**) NOP-1 (within the black box) bound to the GAF domain at binding site III, taken from the merged clusters Cl 1–4 (Fig. 4). Circles indicate the three potential binding sites of the peptide as in panel (**a**). The colour scale of the electrostatic potentials ranges from −3.0 (red) to + 3.0 (blue) *k*_B_*T*/e; the potentials were computed with the Adaptive Poisson-Boltzmann Solver (APBS)^36^. The view of NOP-1 is rotated by 180°, depicting the binding interface with the GAF dimer.

To validate the predictions of the interaction sites, we mutated the residues with the highest frequency of hydrogen bond formation (region I: E177, E178, E246, D283; region II: E190, E204; region III: E152, E169; Fig. 3a) to alanine and probed for NOP-1/GAF dimer interactions *in vitro*. AtETR1^1–307^ variants II (E190A, E204A) and III (E152A, E169A) showed no binding of NOP-1 in the MST experiments (Fig. 3b). In contrast, AtETR1^1–307^ variant I (E177A, E178A, E246A, D283A) interacted with NOP-1 with a similar affinity (*K*_d_ = 128 ± 65 nM) as the unmutated AtETR1^1–307^ (*K*_d_ = 104 ± 24 nM), but with a smaller change in the relative normalized fluorescence (∆*F*_norm_). This is probably due to an increased net electric charge of the variant I and the related change in its hydration sphere, which ultimately influence both temperature-induced fluorescence jump and thermophoresis, and yet do not prevent NOP-1 from binding to the fluorescently labelled protein. Altogether, these results eliminate region I as a NOP-1 interaction site, however they do not clarify the roles of regions II and III in the NOP-1 binding.

To obtain more insights, we performed intrinsic fluorescence quenching experiments. Initially, we mutated two tryptophan residues in the AtETR1 GAF domain (W265 and W288) to phenylalanine to reduce background noise by natural tryptophan residues. The third tryptophan (W182) is located in the interior of the GAF domain and might be important for its structural integrity; hence, we left it unchanged resulting in the AtETR1^1–307^-W265F-W288F construct. This variant was used as reference for individually introducing a tryptophan fluorescence reporter in close proximity of each predicted binding region (Fig. 3a). We then monitored intrinsic tryptophan fluorescence of four Trp-mutants (plus reference variant) in the presence of NOP-1 and found the largest quenching effect in the case of AtETR1^1–307^-M148W-W265F-W288F – a variant with a tryptophan reporter (M148W) located in binding region III (Fig. 3c and Supplementary Fig. S8). When placing the Trp reporter at a more distant position (T161W) to the proposed binding motif at site III, no significant quenching was observed, emphasizing that the NOP-1 inhibitory peptide binds in close proximity to acidic residues E152 and E169 in region III. In addition, the electrostatic potentials mapped onto the molecular surfaces of the GAF dimer and NOP-1 show a strong complementarity at site III, which supports a potential binding motif of NOP-1 at this site (Fig. 3d).

**Figure 4:**
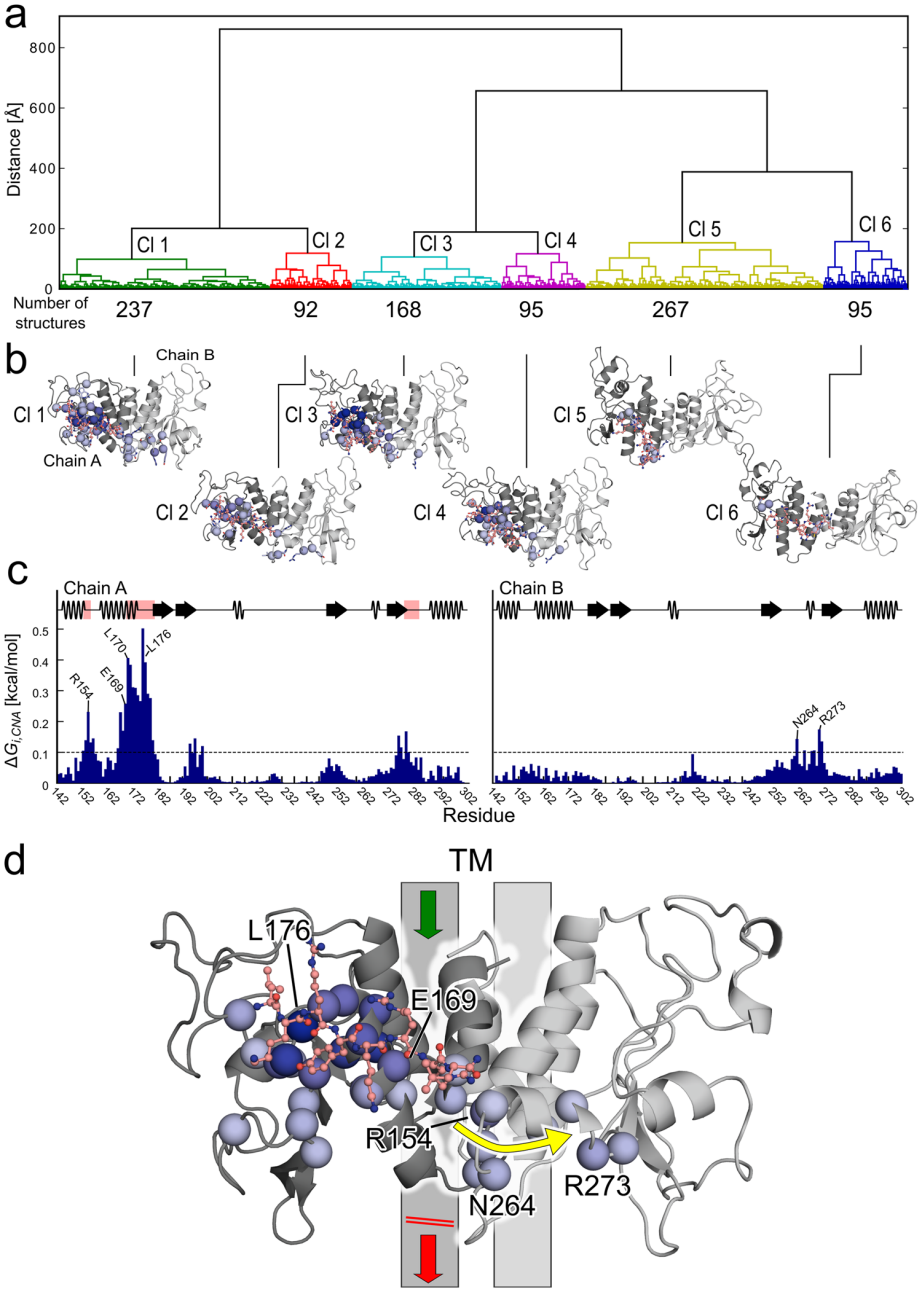
Influence of NOP-1 binding on the structural stability of the AtETR1 GAF dimer. (**a**) The dendrogram shows the clustering of 954 NOP-1 configurations bound at site III of the GAF dimer model (see Fig. 2). Hierarchical clustering was performed using the all-atom RMSD of NOP-1 as distance metric and Ward’s minimum variance algorithm. The dendrogram was cut at a distance threshold *δ*(*c*_1_, *c*_2_) = 160 Å resulting in six clusters (Cl 1–6). *δ*(*c*_1_, *c*_2_) is the square-root of the change in total sum of squares resulting from the fusion of clusters *c*_1_ and *c*_2_.^40,41^ (**b**) CNA was applied on each cluster separately, and residues with ∆*G*_*i*,CNA_ above a threshold of 0.1 kcal mol^−1^ are depicted as spheres on the GAF dimer of each cluster centroid^45^. Blue colors reflect predicted ∆*G*_*i*,CNA_ values, with darker colors indicating larger values. (**c**) The histogram shows the per-residue ∆*G*_*i*,CNA_ of the merged clusters Cl 1–4. The dashed line at 0.1 kcal mol^−1^ indicates the threshold above which residues are considered perturbed, and pink colors highlight the region where NOP-1 binds. (**d**) Same information as shown in (**c**) for the merged clusters Cl 1–4 with NOP-1 bound at site III (salmon). The yellow arrow indicates how the perturbation upon removal of NOP-1 influences residues in chain B. The grey bars indicate connections to the transmembrane (TM) domain and dimerization domain. Due to the increased structural stability of the GAF dimer upon NOP-1 binding, we speculate that the transmission of a signal, arising from ethylene binding to the TM domain of AtETR1, to domains C-terminal of the GAF domain is hampered.

To probe a potential influence of NOP-1 binding on the structural stability of the GAF dimer, we used an ensemble-based perturbation approach^37^ integrated into a method for analysing biomolecular rigidity and flexibility^38^. Initially, we clustered snapshots from the 15 MD simulations of free NOP-1 diffusion, in which NOP-1 binds to binding site III of the GAF domain on chain A (Fig. 4a,b), in order to combine similar configurations of bound NOP-1. Comparing the GAF dimer with and without bound NOP-1 for clusters 1–4 (which cover ~60% of all snapshots) revealed an increase in structural stability upon NOP-1 binding for about 60% of the residues (Fig. 4c). The largest ∆*G*_i_,_CNA_ were found for the loop region (A175–A180) and residues in the neighbouring helix (L167–L174) of the NOP-1-binding domain (Fig. 4c,d), with a maximal ∆*G*_i_,_CNA_ = 0.5 kcal mol^−1^ for residue L176. Notably, even residues up to 20 Å away from the binding site III were influenced by NOP-1 binding, with E273 being the most distant one located in the other domain (Fig. 4c,d). The affected residues form a narrow pathway running across the dimer interface and extending into the other domain. Root mean square fluctuations (RMSF), a measure for atomic mobility, averaged over all MD simulations of the GAF dimer with NOP-1, are smaller by up to ~2 Å compared to MD simulations of the GAF dimer alone in regions distant to binding site III (residues 201–207 and 267–276; Supplementary Fig. S9); these regions coincide with those of higher structural stability identified by the rigidity and flexibility analysis (Fig. 4c,d). Thus, both independent approaches mutually corroborate each other. As the GAF dimer is rotationally symmetric, such an influence will also be felt *vice versa* if NOP-1 binds to the other domain. As a consequence, we speculate that due to the increased structural stability of the GAF dimer, the transmission of a signal, arising from ethylene binding to the TM domain of AtETR1, to domains C-terminal of the GAF domain is hampered (Fig. 4d). The structural stabilization does not contradict the observed Trp fluorescence quenching of the M148W mutant upon NOP-1 binding. We believe a positive charge of NOP-1 in close vicinity of W148 outweighs the positive effect that packing stabilization might have on the fluorescence intensity and results in the overall fluorescence quenching.

In summary, we have shown that the archetypical ripening inhibitory peptide NOP-1 interacts with the GAF domain of the plant ethylene receptor AtETR1 at helices of the dimeric interface. As a result, signal transmission from the TM domain of AtETR1 to the histidine kinase or receiver domains may be hampered, which may explain how NOP-1 inhibits ripening. While currently a full understanding of the AtETR1 signal transduction is hindered by the lack of a complete atomistic structure, our speculation is supported in that for a related histidine kinase^39^ such signal transmission involved TM helix movements that are predicted in computational models to modulate the structural dynamics of the cytoplasmic domains. The predominant predicted binding mode involves primarily residues at the C-terminus of NOP-1, which may explain why the extension of NOP-1 at the N-terminus resulting in NIP-1, N30P, and N41P did not interfere with binding. Hence, this peptide part may be used to further optimize binding, stability, and applicability.

## Materials and Methods

### Inhibitory peptides

C-terminally amidated peptides NOP-1 (LKRYKRRL-NH_2_), NIP-1 (AFPKGKENLASVLKRYKRRL-NH_2_), N30P (GRTGTAAGDVAFPKGKENLASVLKRYKRRL-NH_2_) and N41P (KDVEMAISSRKGRTGTAAGDVAFPKGKENLASVLKRYKRRL-NH_2_) were purchased from GenScript as lyophilized trifluoacetate (TFA) salts with > 98% HPLC purity and stored at −20 °C. After dissolving a white peptide powder in a buffer of choice, peptide concentration in the resulting solution was determined spectroscopically from absorbance at 280 nm and the calculated molar attenuation coefficient (ProtParam)^42^.

### Molecular cloning

All truncated AtETR1 constructs and AtETR1^1–307^ mutants were prepared in pTEV-16b vector backbone^43^, a modified version of pET-16b (Novagen, Darmstadt, Germany) containing the N-terminal decahistidine-tag followed by a linker (SSGH) and a tobacco etch virus (TEV) protease cleavage site (ENLYFQG; instead of a Factor Xa cleavage site in pET-16b). The new constructs were made by using a two-fragment PCR approach^44^ starting from the expression plasmid pTEV-16b-AtETR1 that contains the full-length *Arabidopsis thaliana* ethylene receptor 1 (AtETR1) cDNA. In short, the mutagenesis PCR primers were designed in either PCRdesign or AAscan program^45^ with a 21-nucleotides overlap for a mutagenesis primer pair. Each fragment was amplified in a PCR with Phusion or Q5 high-fidelity DNA polymerase (both from New England BioLabs) or purchased from Integrated DNA Technologies as a gBlocks gene fragment. A pair of fragments was combined into the target plasmid in Gibson assembly^46^, as described in our earlier report^44^. A detailed overview of the molecular cloning as well as the sequences of primers and gene fragments are given in Supplementary Tables S2–S4. The target constructs were verified by sequencing at SEQLAB Sequence Laboratories Göttingen or at the Biological-Medical Research Centre (BMFZ) of the Heinrich Heine University Düsseldorf.

### Expression and purification of AtETR1, its C-terminally truncated constructs and AtETR1^1–307^ mutants

For production of AtETR1 and its variants containing the transmembrane domain, we slightly modified our previous protocol^27^. In brief, the chemically competent *E*. *coli* C43 (DE3) (Lucigen Corporation) cells were transformed with the corresponding pTEV-16b expression plasmid. Transformants were precultured overnight in 2YT medium [16 g L^−1^ peptone, 10 g L^−1^ yeast extract and 5 g L^−1^ NaCl] with 100 µg mL^−1^ ampicillin at 30 °C. Typically, 30 mL preculture was diluted in 500 mL 2YT medium containing 100 µg mL^−1^ ampicillin in a 1-L baffled flask. Cultures were incubated at 30 °C while shaking at 180 rpm. The cells were grown to an optical density at 600 nm (*OD*_600_) between 0.8 and 1.0 and induced with 0.5 mM isopropyl β-d-1-thiogalactopyranoside (IPTG). After incubation for additional 5 h, cells were spun down at 7,500 *g* for 15 min at 4 °C, flash-frozen in liquid nitrogen and stored at −20 °C. If not stated otherwise, all further purification steps were done on ice or at 4 °C. Cell pellets thawed on ice were resuspended by vortexing in ice-cold lysis buffer 1 [pH 8.0, 140 mM NaCl, 2.7 mM KCl, 10 mM Na_2_HPO_4_, 1.8 mM KH_2_PO_4_, 100 g L^−1^ glycerol, 20 mg L^−1^ phenylmethylsulfonyl fluoride (PMSF) and 10 mg L^−1^ DNase I (PanReac AppliChem); 5 mL lysis buffer per 1 g cells] and broken with Constants Cell Disruption System (Constant Systems) at 2.4 kbar and 5 °C. Cell debris and inclusion bodies were removed by centrifugation at 14,000 *g* for 30 min. The supernatant was centrifuged further at 40,000 *g* for 30 min, the resulting pellet was washed with the lysis buffer and centrifuged again at 34,000 *g* for 60 min to isolate cell membranes. Membrane pellets were used immediately in further purification or flash-frozen in liquid nitrogen and stored at −80 °C. To isolate the His-tagged proteins, membranes were resuspended with a paint brush in the solubilization buffer [50 mM Tris/HCl, pH 8.0 at 4 °C, 200 mM NaCl, 12 g L^−1^ fos-choline-16 (*n*-hexadecyl-phosphocholine; Glycon Biochemicals), 20 mg L^−1^ PMSF; 10 mL per 1 g membranes] and incubated for 1 h at 4 °C while mixing. Insoluble part was spun down at 200,000 *g* for 30 min and the supernatant was loaded to a 5-mL Ni-NTA HisTrap FF column (GE Healthcare Life Sciences) equilibrated with buffer A1 [50 mM Tris/HCl, pH 8.0 at 4 °C, 200 mM NaCl, 0.15 g L^−1^ fos-choline-16, 20 mg L^−1^ PMSF]. The protein-loaded column was washed with 25 mL buffer A1, followed by 100 mL buffer ATP1 [buffer A1 with additional 50 mM KCl, 20 mM MgCl_2_ and 10 mM adenosine triphosphate (ATP)] to remove copurified chaperone DnaK, 50 mL buffer A and, finally, 50 mL wash buffer [buffer A1 with 50 mM imidazole]. His-tagged proteins were eluted with 25 mL elution buffer 1 [buffer A1 with 250 mM imidazole] and concentrated in a 100-kDa-MWCO Amicon Ultra-15 concentrator (EDM Millipore) to a final volume 2.5 mL. Buffer was exchanged for storage buffer 1 [50 mM Tris/HCl, pH 8.0 at 20 °C, 300 mM NaCl, 0.15 g L^−1^ fos-choline-16, 50 g L^−1^ glycerol] on a desalting PD-10 column (GE Healthcare Life Sciences) and the sample was centrifuged at 200,000 *g* for 30 min. Protein concentration in the supernatant was determined from absorbance measured at 280 nm and a corresponding molar attenuation coefficient computed using the ProtParam tool^42^. Glycerol was added to purified protein samples to final concentration 200 g L^−1^. The samples with glycerol were distributed into 50-µL aliquots in 200-µL PCR tubes, flash-frozen in liquid nitrogen and stored at −80 °C. Purified proteins were analysed in SDS-PAGE followed by colloidal Coomassie staining^47^ or western blotting to PVDF membrane (Amersham, GE Healthcare Life Sciences) and immunodetection with anti-His-HRP monoclonal antibody (Miltenyi Biotech).

### Expression and purification of AtETR1^306–738^

AtETR1^306–738^ was expressed in chemically competent *E*. *coli* BL21 (DE3) Gold cells (Stratagene) additionally transformed with pBB540 and pBB542 plasmids^48^ (a kind gift from Bernd Bukau, Heidelberg University), carrying the genes for chaperones GrpE, ClpB, DnaK, DnaJ, GroEL and GroES. Typically, 500 mL terrific broth (TB) medium (12 g L^−1^ tryptone, 24 g L^−1^ yeast extract, 5 g L^−1^ glycerol, 2.31 g L^−1^ KH_2_PO_4_ and 12.54 g L^−1^ K_2_HPO_4_) with 100 µg mL^−1^ ampicillin, 34 µg mL^−1^ chloramphenicol and 50 µg mL^−1^ spectinomycin in a 1-L baffled flask was inoculated with 1 mL overnight preculture and incubated at 37 °C while shaking at 160 rpm. The bacteria were grown to *OD*_600_ between 1.1 and 1.3, when they were cooled down on ice (5 min incubation), induced with 0.4 mM IPTG and further grown for 18 h at 20 °C. Cells were spun down (15 min, 7,500 *g*), flash-frozen in liquid nitrogen and stored at −20 °C. As already observed for some other AtETR1 constructs without the transmembrane domain (AtETR1-ΔTM)^32^, purified AtETR1^306–738^ precipitated at higher protein concentrations (>1 mg mL^−1^) in our preliminary purification trials. To circumvent this, we used 0.15 g L^−1^ fos-choline-16 in our purification buffers (the same detergent concentration as for the other AtETR1 constructs with the transmembrane domain described in this work). If not stated otherwise, all purification steps were performed at 4 °C or on ice. The frozen cell pellet was thawed on ice, resuspended in lysis buffer 2 [5 mL buffer per 1 g wet cell pellet; 50 mM Tris/HCl, pH 8.5 at 4 °C, 250 mM NaCl, 20 mM imidazole, 2.5 mM dithiothreitol (DTT), cOmplete EDTA-free protease inhibitor cocktail (Roche) and 10 mg L^−1^ DNase I] and lysed in Constants Cell Disruption System at 2.4 kbar and 5 °C. Insoluble cell debris was separated by centrifugation at 200,000 *g* for 30 min, the supernatant was filtered through 0.22-µm syringe filter and loaded on a 5-mL HisTrap HP column (GE Healthcare Life Sciences) equilibrated with buffer A2 (50 mM Tris/HCl, pH 8.5 at 4 °C, 250 mM NaCl, 2.5 mM DTT, 0.15 g L^−1^ fos-choline-16, cOmplete EDTA-free protease inhibitor cocktail). The column was washed with 50 mL buffer A2, followed by 100 mL buffer ATP2 [50 mM Tris/HCl, pH 8.5 at 4 °C, 250 mM NaCl, 2.5 mM DTT, 0.15 g L^−1^ fos-choline-16, 50 mM KCl, 20 mM MgCl_2_ and 10 mM ATP], 50 mL buffer A2 and 75 mL buffer A2 with 100 mM imidazole. Finally, AtETR1^306–738^ was eluted with 50 mL elution buffer 2 (buffer A2 with 250 mM imidazole) and analysed in SDS-PAGE. The fractions containing the target protein were poured, concentrated (10-kDa-MWCO Amicon Ultra-15 concentrator, EDM Millipore) and imidazole removed by buffer exchange on a PD-10 column for storage buffer 2 [50 mM Tris/HCl, pH 8.5 at at 4 °C, 250 mM NaCl, 0.15 g L^−1^ fos-choline-16, 50 g L^−1^ glycerol, 2.5 mM DTT, cOmplete EDTA-free protease inhibitor cocktail]. The protein sample was centrifuged at 200,000 *g* for 30 min to remove potential aggregates. Finally, glycerol concentration in the supernatant was adjusted to 200 g L^−1^, the sample divided into 50-µL aliquots in 200-µL PCR-tubes, flash-frozen in liquid nitrogen and stored at −80 °C.

### Circular dichroism spectroscopy

Peptides and purified protein constructs were characterized in circular dichroism (CD) spectroscopy. For that, peptides were directly dissolved in degassed ultrapure Milli-Q water (Millipore) or degassed and filtered (0.22-µm filter) CD buffer (10 mM KH_2_PO_4_/K_2_HPO_4_, pH 8.0 at 20 °C) and subsequently diluted to 0.10 mg mL^−1^. Original buffer of protein samples was exchanged for the CD buffer on a desalting PD MiniTrap G-25 column (GE Healthcare Life Sciences). Protein and fos-choline-16 concentrations were determined by using a Direct Detect infrared spectrometer (EMD Millipore) and the samples diluted to final protein concentration 0.10–0.20 mg mL^−1^. Fos-choline-16 was added to each blank buffer solution to match detergent concentration in the final protein samples. CD spectra were recorded at room temperature on a J-715 spectropolarimeter (JASCO) using a 1-mm-path-length cylindrical quartz cuvette (Hellma). Each spectrum represents an average of 10 continuous scans (100 nm min^−1^) with response time 0.25 s and bandwidth 1.0 nm. CD spectra of the peptides were analysed using the K2D2 web server^49^ (Supplementary Fig. S3b,c). Secondary structure content of the protein constructs was calculated in programs CDSSTR^50^, CONTIN^51^ and SELCON3^52,53^ from CDPro software package^54^ using the reference protein set SMP50 (Supplementary Fig. S10 and S11).

### Fluorescent labelling

For the microscale thermophoresis binding experiments, the proteins were labelled with thiol-reactive Alexa Fluor^TM^ 488 C_5_ maleimide fluorescent dye (ThermoFisher Scientific). For that, buffer of a concentrated freshly purified protein sample was exchanged on a desalting PD MiniTrap G-25 column resulting in 800 µL protein sample in labelling buffer [50 mM K_2_HPO_4_/KH_2_PO_4_, 300 mM NaCl and 0.15 g L^−1^ fos-choline-16]. 10 mg mL^−1^ Alexa Fluor^TM^ 488 C_5_ maleimide dimethyl sulfoxide (DMSO) solution was added to the protein sample in 3:1 dye:protein molar ratio and incubated in dark for 30 min at 20 °C while mixing slightly. Buffer was exchange for the storage buffer 2 (AtETR1^306–738^) or storage buffer 1 (all other protein constructs) and the sample centrifuged for 30 min at 200,000 *g* and 4 °C. Spectroscopically determined degrees of labelling in the supernatants ranged from 140% to 300% for different AtETR1 constructs. After adjusting glycerol concentration to 200 g L^−1^, the labelled protein samples were divided into 20-µL aliquots in 200-µL PCR tubes, flash-frozen in liquid nitrogen and stored at −80 °C.

### Microscale thermophoresis (MST)

Each inhibitory peptide was dissolved in the binding buffer [50 mM Tris/HCl, pH 8.0 at 20 °C, 300 mM NaCl, 0.15 g L^−1^ fos-choline-16] and serially diluted for MST measurements. Alexa-Fluor^TM^-488-labelled AtETR1 constructs were diluted with the binding buffer to concentration 50 nM and mixed in a 1:1 volume ratio with each member of the peptide dilution series, resulting in 25 nM fluorescently labelled protein in the final 20-µL mixture. The protein–peptide mixtures were centrifuged at 14,000 *g* for 2 min before filling-up standard treated Monolith NT.115 MST glass capillaries (NanoTemper Technologies). Binding interactions were characterized in Monolith NT.115 Blue/Green (NanoTemper Technology) at 23–25 °C without temperature control. Power of the blue LED (excitation wavelength ca 470 nm) was adjusted depending on a degree of fluorescent labelling of each particular construct and fluorescence. Fluorescence in each capillary (emission wavelength 520 nm) was measured for 5 s without heating, then 30 s heating with 80% infrared laser (MST) power followed by 5 s without heating and 25 s delay before measurement of the next capillary. All measurements were run in at least three independent replicates. Data were evaluated from temperature jump (fluorescence signal between 0.5 s and 1.5 s after applying the laser normalized with the fluorescence signal in the last second before applying the laser) and fitted with nonlinear regression to the one-binding-site model^55–57^ in GraphPad Prism version 7.00 for Windows (GraphPad Software, La Jolla California USA). As a negative control, a protein sample was diluted in the denaturation buffer [50 mM Tris/HCl, pH 8.0 at 20 °C, 300 mM NaCl, 0.15 g L^−1^ fos-choline-16, 40 g L^−1^ sodium dodecyl sulfate (SDS) and 40 mM DTT] and the MST measurements were carried out as described above.

### Model building

The model structure of the GAF domain (amino acid 142 to 305 of AtETR1) was predicted using our in-house automated structure prediction pipeline TopModel^31,58^. TopModel is a multi-template meta-approach in which 20 different state-of-the-art threaders (see Supplementary Table S1) are used to detect homologous templates. For each template the Topmodel-Score^59^ to the native structure, a measure of structural similarity, is predicted using deep neural networks. These networks use alignment features, PSIPRED^60^ secondary structure agreement, threading scores from individual threaders, model quality predicted by TopScore (D. Mulnaes, H. Gohlke, unpublished results; see also below for details), and structural consensus as input. Based on the neural network predictions, false positive templates are removed, consensus alignments are calculated, and the templates are ranked according to predicted TopModel-Scores. To sample different alignments, TopModel makes an ensemble of multiple sequence alignments (MSAs) using all combinations of the top five templates and eight different sequence and structure alignment programs (see Supplementary Table S1). These MSAs are used to generate 3D models of the GAF domain using Modeller9^61^ and the template structures. Loops without template were refined using the DOPE potential^62^ and secondary structure restraints based on PSIPRED predictions. The generated models were ranked with TopScore, and the highest ranked model for each template combination was selected for model combination and refinement. The selected models are refined with ModRefiner^63^ and scored with TopScore. Based on TopScore predictions, regions with errors are removed and the remaining regions used as templates to construct meta-models. Two iterations of this refinement and model combination is performed, after which the best scoring model according to TopScore is selected as the final model of the GAF domain.

The correctness of the model is measured by TopScore as the predicted global and local lDDT score compared to the native structure. The lDDT score compares all intra-molecular heavy-atom distances within two structures and, thus, is superposition-free. Two models are considered completely different if all distances deviate by more than 4 Å, and completely identical if all distances deviate by less than 0.5 Å. Since the native structure is unknown in our case, the score is predicted by a deep neural network which uses multiple sources of information as input. These include knowledge-based angle, distance and contact potentials, residue stereochemistry, atom clashes, model clustering, and agreement between features predicted from the sequence and measured in the model, such as secondary structure, solvent accessibility, and residue contacts. The deep neural network was trained on a large data-set of 660 protein targets totaling over 133,000 models and over 19·10^6^ residues.

### Molecular dynamics (MD) simulations

The model structure of the GAF domain (amino acid 142 to 305 of AtETR1) and the linear forms (*ϕ* = *ψ* = 180°) of NOP-1, NIP-1, N30P, and N41P with a C-terminal amino (NHE)-cap served as input structures for MD simulations. For receptor–peptide interaction studies, NOP-1 was randomly placed next to the GAF dimer with a minimum distance of 8 Å using the software package PackMol^64^; fifteen representative systems were generated that way. The solutes were placed in a truncated octahedral box of TIP3P^65^ water leaving a distance of at least 11 Å between the protein and the solvation box boundaries, and Na^+^ and Cl^−^ ions were added to reach a final salt concentration of 0.15 M. MD simulations were performed with the ff14SB force field^66^. Hydrogen mass repartitioning was used, allowing a time step of 4 fs^67^. Further parameters for system preparation, thermalization, and production runs are described in Minges *et al*.^68^. In short, each system was prepared performing a conjugate gradient minimization, followed by rising the temperature from 0 K to 300 K (over 100 ps) and adjusting the system density under NPT conditions. Production NVT-MD simulations were performed at 300 K utilizing the Berendsen thermostat^69^, and conformations were saved every 100 ps.

For peptide folding simulations, three independent replicates (initiated by slightly different thermalization temperatures) of 50 µs simulation length were performed for each system. All simulations were performed in implicit solvent using the ff14SBonlysc force field in combination with mbondi3 radii and the GB-Neck2 model^70^ as described by Nguyen *et al*.^28^. In short, after minimization and thermalization, MD simulations were performed with a time step of 4 fs using hydrogen mas*s* repartitioning^67^, temperature control at 300 K with a Langevin thermostat^71^, and a long-range distance cut-off of 999 Å. Conformations were saved every 1 ns.

The trajectories were analysed with respect to secondary structure formation, distribution of NOP-1 around the GAF dimer, and RMSF using *cpptraj*^72^. The DSSP method of Kabsch and Sander^73^ was utilized to calculate secondary structure types of each residue of NOP-1, NIP-1, N30P, and N41P. Values were averaged over all trajectories. For calculating the distribution of NOP-1 around the GAF dimer along the 15 MD simulations of free NOP-1 diffusion, the snapshots were superimposed onto the starting structure of the GAF dimer, a cubic grid with bin size 3 × 250 Å^2^ was placed in the simulation box, and the presence of the centre of mass of NOP-1 within a grid bin was assessed after 100, 500, and 2000 ns of simulation time over all snapshots. The number of hydrogen bonds (and salt bridges) formed between NOP-1 and each residue of the GAF dimer over all trajectories was determined using VMD^74^, where NOP-1 was chosen as donor and the receptor as acceptor molecule. Prior to computing C_α_ atom RMSF, snapshots of either the 15 MD simulations of free NOP-1 diffusion or the three MD simulations of the *apo* GAF dimer were superimposed onto the starting structure of the GAF dimer.

### Tryptophan fluorescence

Steady-state intrinsic fluorescence of the freshly prepared AtETR1^1–307^ Trp-mutants was measured on a LS-55 fluorescence spectrometer (PerkinElmer) using an excitation wavelength 295 nm. In the last protein purification step, the elution buffer 1 was exchanged for the binding buffer on a desalting PD MiniTrap G-25 column. To monitor binding of NOP-1 by fluorescence quenching, each protein sample was diluted with the same buffer to final concentration 1 µM and titrated with a concentrated stock solution of NOP-1 in the binding buffer at room temperature (22 °C) while stirring slowly in a 4-mm Quartz SUPRASIL Macro/Semi-micro cell with a small magnet (PerkinElmer). At the same time, intensity of an emission maximum at 344 nm was recorded as an average of 5 measurements. Fluorescence readings were corrected for the dilution effect. The inner filter effect of NOP-1 was negligible and could be ignored.

### Constraint Network Analysis

To detect changes in biomolecular rigidity and flexibility upon NOP-1 binding, we analysed ensembles of snapshots in the biomolecule’s bound and unbound states in terms of a perturbation approach^37^. First, an ensemble of network topologies is saved every 2 ns from the 15 × 2 µs of independent, unbiased MD simulations of free NOP-1 diffusion around the GAF dimer (see above). From this ensemble of 150,000 conformations, those conformations were extracted that have a hydrogen bond between NOP-1 and the residues E152 or E169, indicative of NOP-1 binding to site III of the GAF dimer; this yielded 954 snapshots for the ground state. The perturbed state is obtained by removing the covalent and non-covalent interactions associated with NOP-1 from each network topology of the ground state. In order to further group similar binding modes of NOP-1, we clustered NOP-1 conformations based on a pairwise all-atom RMDS according to Ward’s method as implemented in SciPy^75^. This resulted in six clusters (see Fig. 4a). Second, altered biomolecular stability due to removal of NOP-1 is quantified in terms of a per-residue decomposition Δ*G*_*i,CNA*_ of the perturbation free energy. Δ*G*_*i,CNA*_ was computed based on rigidity analyses performed with the CNA software package^38^ on the ensembles of network topologies of the ground and perturbed states. Network topologies (containing nodes (atoms) and constraints (covalent and non-covalent interactions)) were constructed with the FIRST (Floppy Inclusions and Rigid Substructure Topography) software (version 6.2)^76^ to which CNA is a front and back end. The strength of hydrogen bonds (including salt bridges) were assigned by the energy *E*_HB_ computed by FIRST^77^. Hydrophobic interactions between carbon or sulfur atoms were taken into account if the distance between these atoms was less than the sum of their van der Waals radii (C: 1.7 Å, S: 1.8 Å) plus *D*_cut_ = 0.25 Å^78^. Non-covalent interactions between NOP-1 and the GAF domain were identified using knowledge-based DrugScore pair potentials^79^.

When CNA was applied on each cluster 1–6 (see above) separately, the clusters 5 and 6 revealed only minor and local altered structural stability of the GAF dimer upon NOP-1 removal (see Fig. 4b) and, thus, were excluded from further analyses. Clusters 1–4 were merged for subsequent analyses. This resulted in a final ensemble of 592 snapshots used as input for CNA. Upon perturbation, the network topologies lose on average 7.5 (=1.3% of all) hydrogen bond constraints and 2.2 (=1.6% of all) hydrophobic tether constraints. About 60% of the residues in the GAF domain show altered stability characteristic, with 9% of the residues having Δ*G*_*i,CNA*_ values > 0.1 kcal mol^−1^ upon removal of NOP-1.

### Electrostatic surface potential

The electrostatic surface potential for the GAF dimer and NOP-1 was calculated using the Adaptive Poisson-Boltzmann Solver (APBS)^36^. The complex structure of the GAF dimer and NOP-1 were first split into their single components. For the APBS calculations, default parameters were used, the temperature of the system was set to 300 K, and the concentration of 1:1 counterions to 0.15 M.

### Data availability statement

The data generated and analysed during the current study are either included in this published article and its Supplementary Information file or available from the corresponding authors on reasonable request.

## Electronic supplementary material

Supplementary Information

## References

[1] Payasi A, Sanwal GG (2010). Ripening of climacteric fruits and their control. J. Food Biochem..

[2] Bleecker AB, Estelle MA, Somerville C, Kende H (1988). Insensitivity to Ethylene Conferred by a Dominant Mutation in *Arabidopsis thaliana*. Science.

[3] Chang C, Kwok S, Bleecker A, Meyerowitz E (1993). *Arabidopsis* ethylene-response gene ETR1: similarity of product to two-component regulators. Science.

[4] Hua J, Chang C, Sun Q, Meyerowitz E (1995). Ethylene insensitivity conferred by *Arabidopsis* ERS gene. Science.

[5] Hua J, Meyerowitz EM (1998). Ethylene Responses Are Negatively Regulated by a Receptor Gene Family in *Arabidopsis thaliana*. Cell.

[6] Hua J (1998). EIN4 and ERS2 Are Members of the Putative Ethylene Receptor Gene Family in Arabidopsis. The Plant Cell.

[7] Grefen C (2008). Subcellular Localization and *In Vivo* Interactions of the *Arabidopsis thaliana* Ethylene Receptor Family Members. Mol. Plant.

[8] Bleecker AB, Kende H (2000). Ethylene: A Gaseous Signal Molecule in Plants. Annu. Rev. Cell Dev. Biol..

[9] Stepanova AN, Ecker JR (2000). Ethylene signaling: from mutants to molecules. Curr. Opin. Plant. Biol..

[10] Kieber JJ, Rothenberg M, Roman G, Feldmann KA, Ecker JR (1993). CTR1, a negative regulator of the ethylene response pathway in Arabidopsis, encodes a member of the Raf family of protein kinases. Cell.

[11] Gao Z (2003). Localization of the Raf-like Kinase CTR1 to the Endoplasmic Reticulum of *Arabidopsis* through Participation in Ethylene Receptor Signaling Complexes. J. Biol. Chem..

[12] Alonso JM, Hirayama T, Roman G, Nourizadeh S, Ecker JR (1999). EIN2, a Bifunctional Transducer of Ethylene and Stress Responses in *Arabidopsis*. Science.

[13] Bisson MMA, Groth G (2015). Targeting Plant Ethylene Responses by Controlling Essential Protein–Protein Interactions in the Ethylene Pathway. Mol. Plant.

[14] Bisson MMA (2016). Peptides interfering with protein-protein interactions in the ethylene signaling pathway delay tomato fruit ripening. Sci. Rep..

[15] Kessenbrock, M. *et al*. Novel Protein-Protein Inhibitor Based Approach to Control Plant Ethylene Responses: Synthetic Peptides for Ripening Control. *Front*. *Plant Sci*. **8**, 10.3389/fpls.2017.01528 (2017). 10.3389/fpls.2017.01528 PMC5591945 28928762

[16] Bisson MMA, Groth G (2011). New paradigm in ethylene signaling: EIN2, the central regulator of the signaling pathway, interacts directly with the upstream receptors. Plant Signal. Behav..

[17] Qiao H, Chang KN, Yazaki J, Ecker JR (2009). Interplay between ethylene, ETP1/ETP2 F-box proteins, and degradation of EIN2 triggers ethylene responses in. Arabidopsis. Genes Dev..

[18] Qiao H (2012). Processing and Subcellular Trafficking of ER-Tethered EIN2 Control Response to Ethylene Gas. Science.

[19] Wen, X. *et al*. Activation of ethylene signaling is mediated by nuclear translocation of the cleaved EIN2 carboxyl terminus. *Cell Res.* **22**, 1613–1616, 10.1038/cr.2012.145 (2012). 10.1038/cr.2012.145 PMC3494400 23070300

[20] Li W (2015). EIN2-directed translational regulation of ethylene signaling in *Arabidopsis*. Cell.

[21] Merchante C (2015). Gene-Specific Translation Regulation Mediated by the Hormone-Signaling Molecule EIN2. Cell.

[22] Xie F, Liu Q, Wen C-K (2006). Receptor Signal Output Mediated by the ETR1 N Terminus Is Primarily Subfamily I Receptor Dependent. Plant Physiol..

[23] Gao Z (2008). Heteromeric Interactions among Ethylene Receptors Mediate Signaling in Arabidopsis. J. Biol. Chem..

[24] Gamble RL, Qu X, Schaller GE (2002). Mutational Analysis of the Ethylene Receptor ETR1. Role of the Histidine Kinase Domain in Dominant Ethylene Insensitivity. Plant Physiol..

[25] Binder, B. M. & Bleecker, A. B. A Model for Ethylene Receptor Function and 1-Methylcyclopropene Action *Acta Hortic*., 177-187, 10.17660/ActaHortic.2003.628.21 (2003).

[26] Binder BM (2004). *Arabidopsis* Seedling Growth Response and Recovery to Ethylene. A Kinetic Analysis. Plant Physiol..

[27] Binder BM, Mortimore LA, Stepanova AN, Ecker JR, Bleecker AB (2004). Short-Term Growth Responses to Ethylene in *Arabidopsis* Seedlings Are EIN3/EIL1 Independent. Plant Physiol..

[28] Nguyen H, Maier J, Huang H, Perrone V, Simmerling C (2014). Folding simulations for proteins with diverse topologies are accessible in days with a physics-based force field and implicit solvent. J. Am. Chem. Soc..

[29] Maffucci I, Contini A (2016). An Updated Test of AMBER Force Fields and Implicit Solvent Models in Predicting the Secondary Structure of Helical, beta-Hairpin, and Intrinsically Disordered Peptides. J. Chem. Theory Comput..

[30] Ward JJ, Sodhi JS, McGuffin LJ, Buxton BF, Jones DT (2004). Prediction and functional analysis of native disorder in proteins from the three kingdoms of life. J. Mol. Biol..

[31] Gohlke H (2013). Binding region of alanopine dehydrogenase predicted by unbiased molecular dynamics simulations of ligand diffusion. J. Chem. Inf. Model..

[32] Mayerhofer H (2015). Structural Model of the Cytosolic Domain of the Plant Ethylene Receptor 1 (ETR1). J. Biol. Chem..

[33] Dominguez C, Boelens R, Bonvin AM (2003). HADDOCK: a protein-protein docking approach based on biochemical or biophysical information. J. Am. Chem. Soc..

[34] Ahmad M, Gu W, Helms V (2008). Mechanism of fast peptide recognition by SH3 domains. Angew. Chem. Int. Ed. Engl..

[35] Zwier MC (2016). Efficient Atomistic Simulation of Pathways and Calculation of Rate Constants for a Protein-Peptide Binding Process: Application to the MDM2 Protein and an Intrinsically Disordered p53 Peptide. J. Phys. Chem. Lett..

[36] Jurrus E (2018). Improvements to the APBS biomolecular solvation software suite. Protein Sci..

[37] Pfleger C (2017). Ensemble- and rigidity theory-based perturbation approach to analyze dynamic allostery. J. Chem. Theory Comput..

[38] Pfleger C, Rathi PC, Klein DL, Radestock S, Gohlke H (2013). Constraint Network Analysis (CNA): A Python Software Package for Efficiently Linking Biomacromolecular Structure, Flexibility, (Thermo-)Stability, and Function. J. Chem. Inf. Model..

[39] Lemmin T, Soto CS, Clinthorne G, DeGrado WF, Dal Peraro M (2013). Assembly of the transmembrane domain of E. coli PhoQ histidine kinase: implications for signal transduction from molecular simulations. PLoS Comput. Biol..

[40] Kaufman, L. & Rousseeuw, P. J. Finding Groups in Data: An Introduction to Cluster Analysis. Vol. 344 (John Wiley & Sons, 2009).

[41] Legendre, P. & Legendre, L. F. J. Numerical ecology. Vol. 24 (Elsevier, 2012).

[42] Gasteiger, E. *et al*. In *The Proteomics Protocols Handbook* (ed John M. Walker) 571-607 (Humana Press, 2005).

[43] Classen E, Groth G (2012). Cloning, expression and purification of orthologous membrane proteins: a general protocol for preparation of the histidine sensor kinase ETR1 from different species. Mol. Membr. Biol..

[44] Heydenreich FM (2017). High-throughput mutagenesis using a two-fragment PCR approach. Sci. Rep..

[45] Sun D (2013). AAscan, PCRdesign and MutantChecker: A Suite of Programs for Primer Design and Sequence Analysis for High-Throughput Scanning Mutagenesis. PLoS One.

[46] Gibson DG (2009). Enzymatic assembly of DNA molecules up to several hundred kilobases. Nat. Methods.

[47] Kang D-H, Gho Y-S, Suh M-K, Kang C-H (2002). Highly Sensitive and Fast Protein Detection with Coomassie Brilliant Blue in Sodium Dodecyl Sulfate-Polyacrylamide GelElectrophoresis. Bull. Korean Chem. Soc..

[48] de Marco A, Deuerling E, Mogk A, Tomoyasu T, Bukau B (2007). Chaperone-based procedure to increase yields of soluble recombinant proteins produced in E. coli. BMC Biotechnol..

[49] Perez-Iratxeta C, Andrade-Navarro MA (2008). K2D2: estimation of protein secondary structure from circular dichroism spectra. BMC Struct Biol.

[50] Johnson, W. C. Analyzing protein circular dichroism spectra for accurate secondary structures. *Proteins: Struct., Funct., Bioinf.* **35**, 307–312, https://doi.org10.1002/(SICI)1097-0134(19990515)35:3<307::AID-PROT4>3.0.CO;2-3 (1999). 10328265

[51] Provencher SW, Gloeckner J (1981). Estimation of globular protein secondary structure from circular dichroism. Biochemistry.

[52] Sreerama N, Woody RW (1993). A Self-Consistent Method for the Analysis of Protein Secondary Structure from Circular Dichroism. Anal. Biochem..

[53] Sreerama N, Venyaminov SYU, Woody RW (1999). Estimation of the number of α-helical and β-strand segments in proteins using circular dichroism spectroscopy. Protein Sci..

[54] Sreerama N, Woody RW (2000). Estimation of Protein Secondary Structure from Circular Dichroism Spectra: Comparison of CONTIN, SELCON, and CDSSTR Methods with an Expanded Reference Set. Anal. Biochem..

[55] Wienken CJ, Baaske P, Rothbauer U, Braun D, Duhr S (2010). Protein-binding assays in biological liquids using microscale thermophoresis. Nat. Commun..

[56] Seidel SAI (2013). Microscale thermophoresis quantifies biomolecular interactions under previously challenging conditions. Methods.

[57] Jerabek-Willemsen M, Wienken CJ, Braun D, Baaske P, Duhr S (2011). Molecular Interaction Studies Using Microscale Thermophoresis. Assay Drug Dev. Technol..

[58] Widderich N (2014). Molecular dynamics simulations and structure-guided mutagenesis provide insight into the architecture of the catalytic core of the ectoine hydroxylase. J. Mol. Biol..

[59] Zhang Y, Skolnick J (2005). TM-align: a protein structure alignment algorithm based on the TM-score. Nucleic Acids Res..

[60] McGuffin LJ, Bryson K, Jones DT (2000). The PSIPRED protein structure prediction server. Bioinformatics.

[61] Webb B, Sali A (2014). Comparative protein structure modeling using Modeller. Curr. Protoc. Bioinformatics.

[62] Shen MY, Sali A (2006). Statistical potential for assessment and prediction of protein structures. Protein Sci..

[63] Xu D, Zhang Y (2011). Improving the physical realism and structural accuracy of protein models by a two-step atomic-level energy minimization. Biophys. J..

[64] Martinez L, Andrade R, Birgin EG, Martinez JM (2009). PACKMOL: a package for building initial configurations for molecular dynamics simulations. J. Comput. Chem..

[65] Jorgensen WL, Chandrasekhar J, Madura JD, Impey RW, Klein ML (1983). Comparison of Simple Potential Functions for Simulating Liquid Water. J. Chem. Phys..

[66] Maier JA (2015). ff14SB: Improving the Accuracy of Protein Side Chain and Backbone Parameters from ff99SB. J. Chem. Theory Comput..

[67] Hopkins CW, Le Grand S, Walker RC, Roitberg AE (2015). Long-Time-Step Molecular Dynamics through Hydrogen Mass Repartitioning. J. Chem. Theory Comput..

[68] Minges A (2017). Structural intermediates and directionality of the swiveling motion of Pyruvate Phosphate Dikinase. Sci. Rep..

[69] Berendsen HJC, Postma JPM, Vangunsteren WF, Dinola A, Haak JR (1984). Molecular-Dynamics with Coupling to an External Bath. J. Chem. Phys..

[70] Nguyen H, Roe DR, Simmerling C (2013). Improved Generalized Born Solvent Model Parameters for Protein Simulations. J. Chem. Theory Comput..

[71] Larini L, Mannella R, Leporini D (2007). Langevin stabilization of molecular-dynamics simulations of polymers by means of quasisymplectic algorithms. J. Chem. Phys..

[72] Roe DR, Cheatham TE (2013). PTRAJ and CPPTRAJ: Software for Processing and Analysis of Molecular Dynamics Trajectory Data. J. Chem. Theory Comput..

[73] Kabsch, W. & Sander, C. Dictionary of protein secondary structure: pattern recognition of hydrogen-bonded and geometrical features. *Biopolymers* **22**, 2577–2637, 10.1002/bip.360221211 (1983). 10.1002/bip.360221211 6667333

[74] Humphrey W, Dalke A, Schulten K (1996). VMD: visual molecular dynamics. J. Mol. Graph..

[75] Jones, E. *SciPy: Open Source Scientific Tools for Python*,http://www.scipy.org/ (2001).

[76] Jacobs DJ, Rader AJ, Kuhn LA, Thorpe MF (2001). Protein flexibility predictions using graph theory. Proteins.

[77] Dahiyat BI, Gordon DB, Mayo SL (1997). Automated design of the surface positions of protein helices. Protein Sci..

[78] Rader AJ, Hespenheide BM, Kuhn LA, Thorpe MF (2002). Protein unfolding: rigidity lost. Proc. Natl. Acad. Sci. USA.

[79] Gohlke H, Hendlich M, Klebe G (2000). Knowledge-based scoring function to predict protein-ligand interactions. J. Mol. Biol..

