# Supplementary material for: Recognition motif and mechanism of ripening inhibitory peptides in plant hormone receptor ETR1
Source: Sci Rep. 2018 Mar 1;8:3890. doi: 10.1038/s41598-018-21952-3 (PMC5832771; doi:10.1038/s41598-018-21952-3)
Supplement: Supplementary file 1 — Supplementary Information [file 41598_2018_21952_MOESM1_ESM.pdf]

Supplementary Information

Recognition motif and mechanism of ripening inhibitory peptides in plant hormone receptor ETR1

Dalibor Milić[1,†,‡], Markus Dick[2,†,§] , Daniel Mulnaes[2], Christopher Pfleger[2], Anna Kinnen[1], Holger Gohlke[2,3,*] and Georg Groth[1,*]

[1]Institute of Biochemical Plant Physiology and Bioeconomy Science Center (BioSC), Heinrich Heine University Düsseldorf, Düsseldorf, Germany

[2]Institute of Pharmaceutical and Medicinal Chemistry and Bioeconomy Science Center (BioSC), Heinrich Heine University Düsseldorf, Düsseldorf, Germany

[3]John von Neumann Institute for Computing (NIC), Jülich Supercomputing Centre (JSC) & Institute for Complex Systems - Structural Biochemistry (ICS 6), Forschungszentrum Jülich GmbH, Jülich, Germany

[†]D.M. and M.D. contributed equally to this work.
[‡]D.M. present address: Department of Structural and Computational Biology, Max F. Perutz Laboratories, University of Vienna, Vienna Biocenter, Vienna, Austria
[§]M.D. present address: Division of Chemistry and Chemical Engineering, California Institute of Technology, Pasadena, California, USA

*Correspondence should be addressed to G.G., e-mail: georg.groth@hhu.de or H.G., e-mail: gohlke@hhu.de, h.gohlke@fz-juelich.de

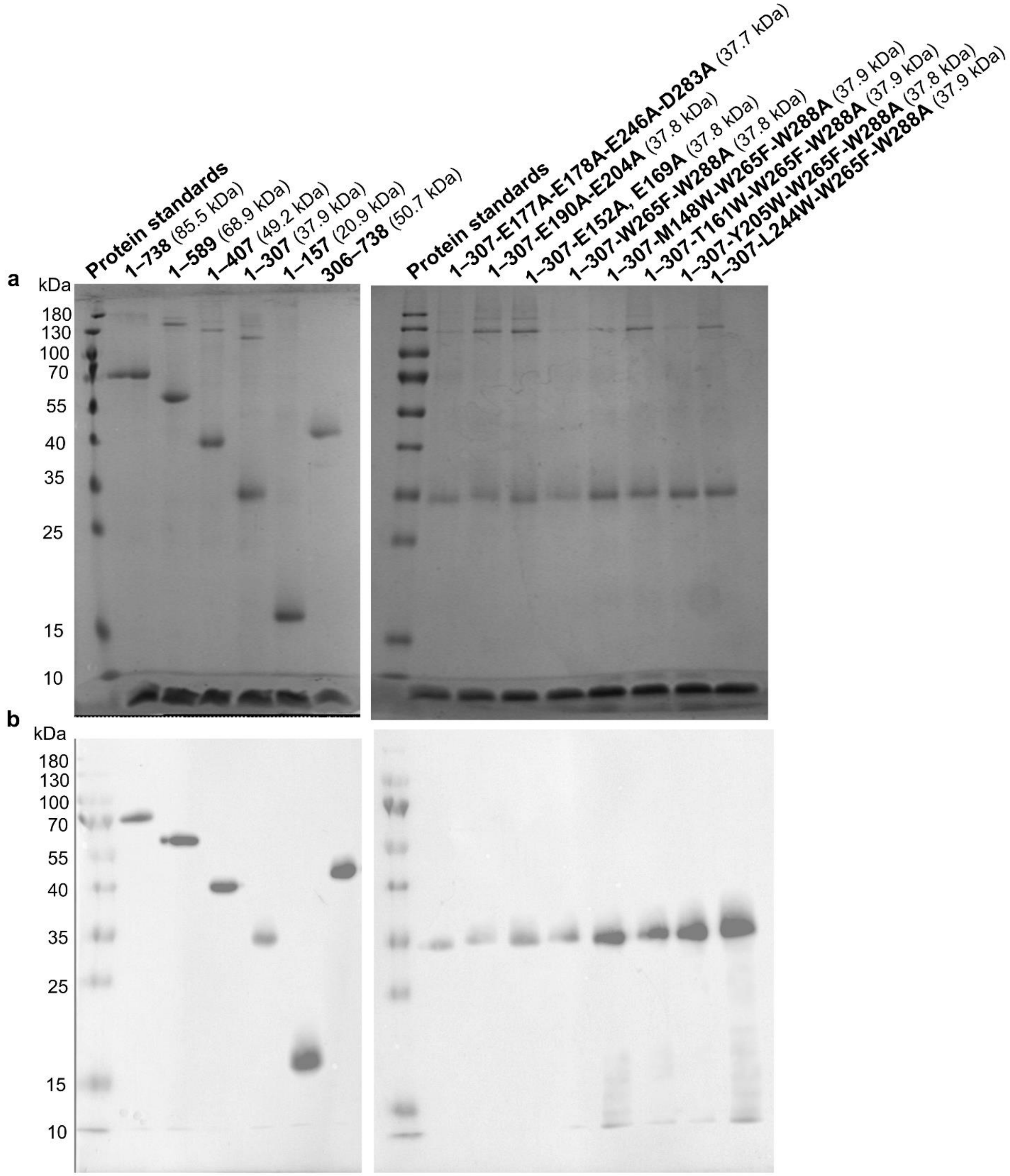

Supplementary Figure S1 | SDS-PAGE of the truncated His-tagged AtETR1 constructs. (**a**) Coomassie-stained gels. (**b**) Western blotting with the anti-His antibody. Molecular weight of each construct is given in parentheses. The gel bands below the dye front are artefacts due to fos-choline-16 – a detergent used in purification.

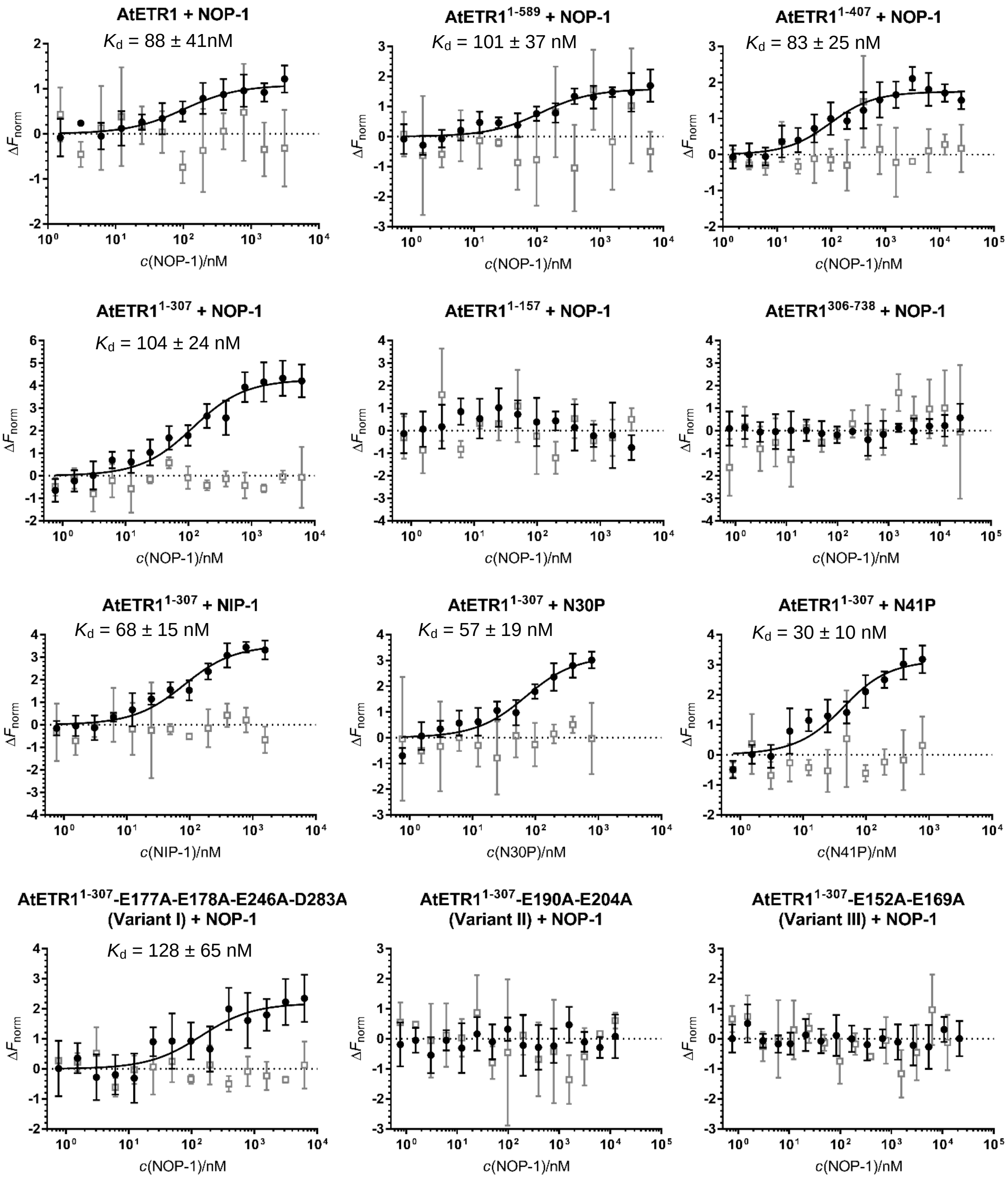

Supplementary Figure S2 | Microscale thermophoresis (MST) interaction studies of the fluorescently labelled AtETR1 mutants with peptides NOP-1, NIP-1, N30P, and N41P. Relative normalized fluorescence (ΔF_{norm}; ●) was fitted to the one-binding-site model, and the corresponding K_d value is given for each binding curve. Data for the chemically denatured proteins (□) are given for comparison.

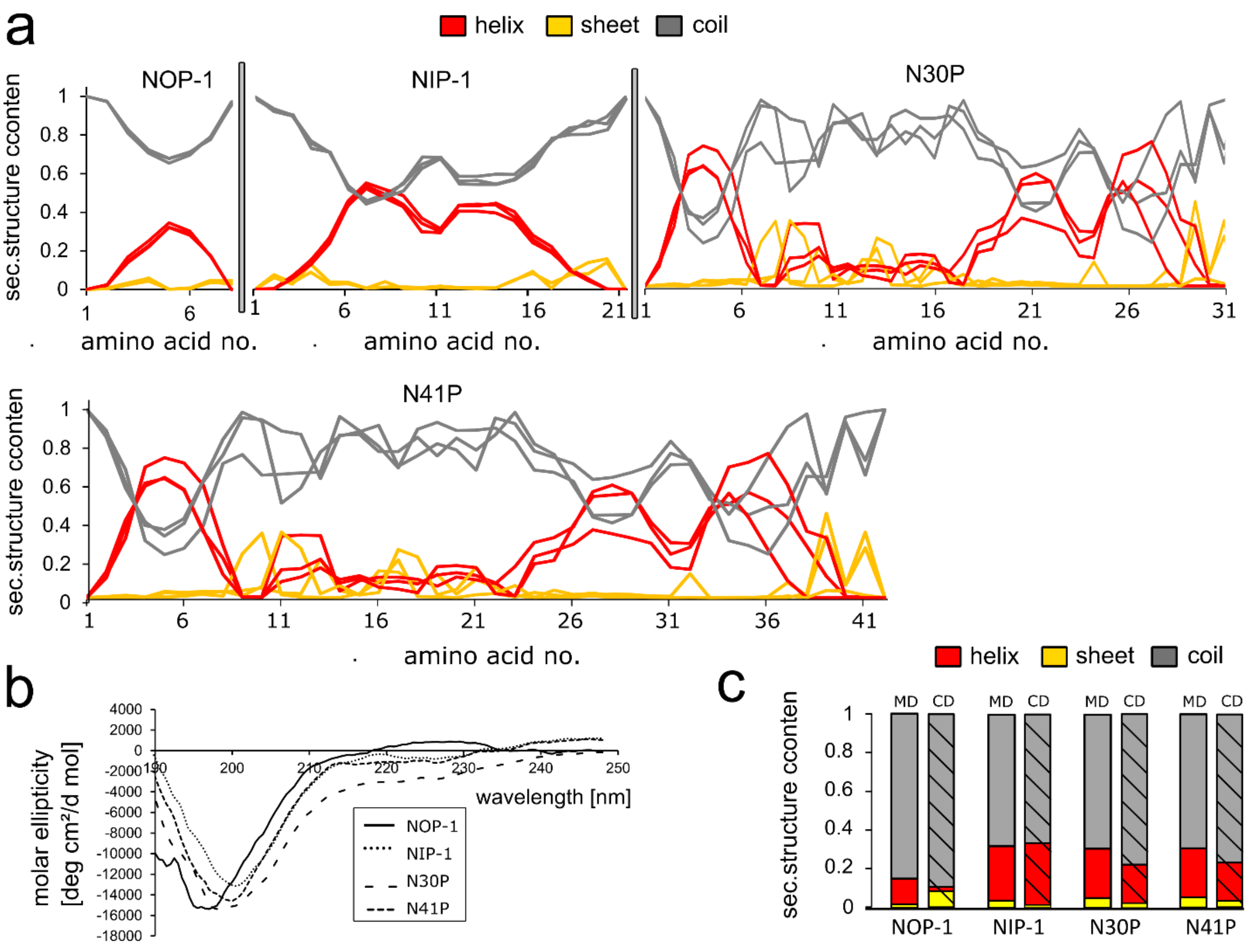

Supplementary Figure S3 | Secondary structure of the four EIN2-derived peptides. (**a**) Residue-wise secondary structure prediction based on three MD simulations for each system in implicit solvent. The secondary structure content was calculated by DSSP [1] as an average over all snapshots in 50 µs of MD simulations. (**b**) Far UV-CD scan for NOP-1, NIP-1, N30P and N41P. (**c**) Comparison of secondary structure contents predicted by MD simulations (mean over all residues and three MD simulations) and computed from CD data (using the K2D2 web server[2]).

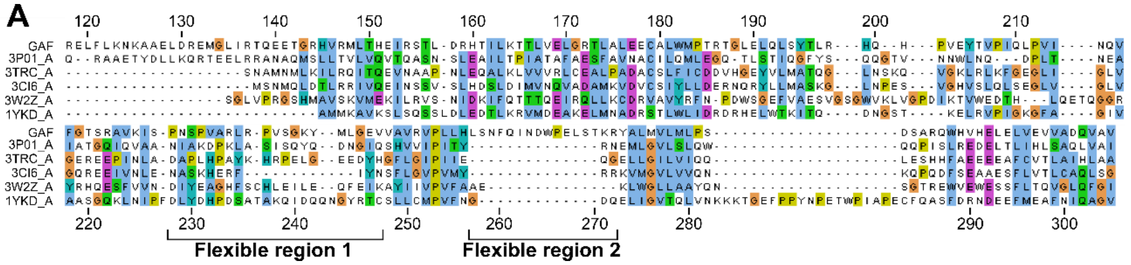

Supplementary Figure S4 | Sequence alignment between the AtETR1 GAF domain (residues 118–304) and five template sequences used to predict the structure. The flexible regions 1 (residues 228–247) and 2 (residues 257–272) with the highest degree of inaccuracy in the final GAF domain model are indicated in brackets. The alignment was calculated using the in-house meta-alignment tool TopAligner as a consensus between alignments calculated by eight different state-of-the-art multiple alignment programs. The alignment programs used are given in **Supplementary Table 1**.

Supplementary Table S1 | TopModel methods used for threading, alignment and model quality assessment of the GAF domain model

Threading	Alignment	Quality Assessment
DeltaBLAST [3]	TCOFFEE [4] [1]	PROCHECK [5]
HMMER3 [6]	MAFFT7 [7]	MolProbity [8]
HHBlits [9]	MergeAlign2 [10]	ANOLEA [11]
HHSearch[12]	SAlign [13]	ProSA2003 [14]
FFAS03 [15]	PROMALS3D [16]	DOPE [17]
SPARKSX [18]	FORMATT [19]	GOAP [20]
RAPTORX [21]	MUSTANG [22]	ModFOLDClust2 [23]
LOMETS [24] [2]	3DCOMB [25]	PCONS [26]
pGenThreader [27]		SPICKER [28]
pDomThreader [27]		QMEAN6 [29]
FASTA [30]		PROQ2 [31]
SAMT2K [32]		SELECTPRO [33]

[1] The following programs are used within the TCOFFEE suite as the default methods for calculating alignments: ClustalW [34], POA [35], MUSCLE [36], ProbA [37], PCMA [38], ProbCons [39], DiAlign [40], SAP [41], and TM-Align [42].

[2] The LOMETS software includes the algorithms PPAS, wPPAS, dPPAS, wdPPAS, PPAS2, dPPAS2, Env-PPAS, MUSTER, and wMUSTER.

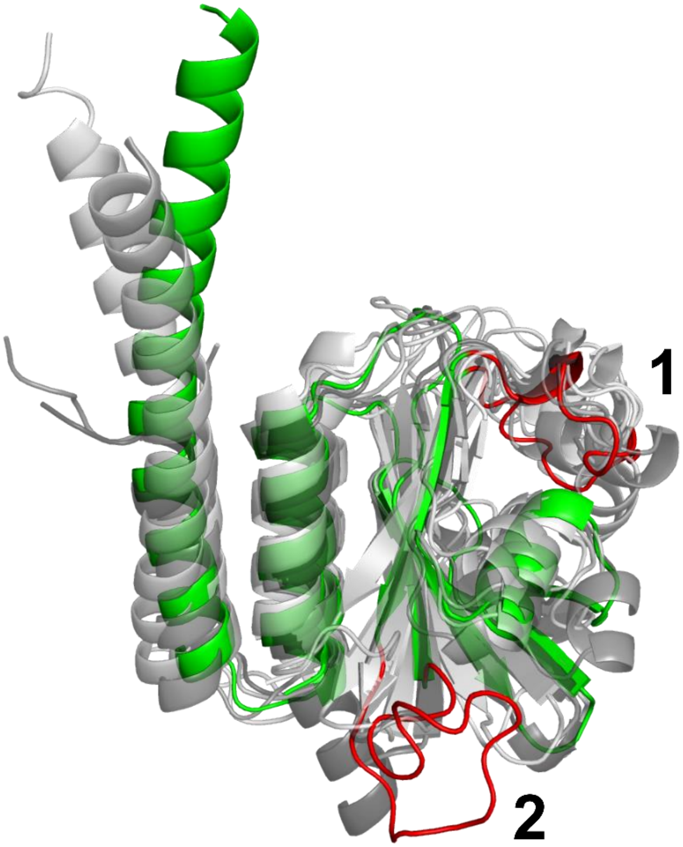

Supplementary Figure S5 | Structural alignment between the final GAF domain model and the five templates used to predict the structure. The flexible regions 1 (residues 228–247) and 2 (residues 257–272) with the highest degree of inaccuracy are indicated in red.

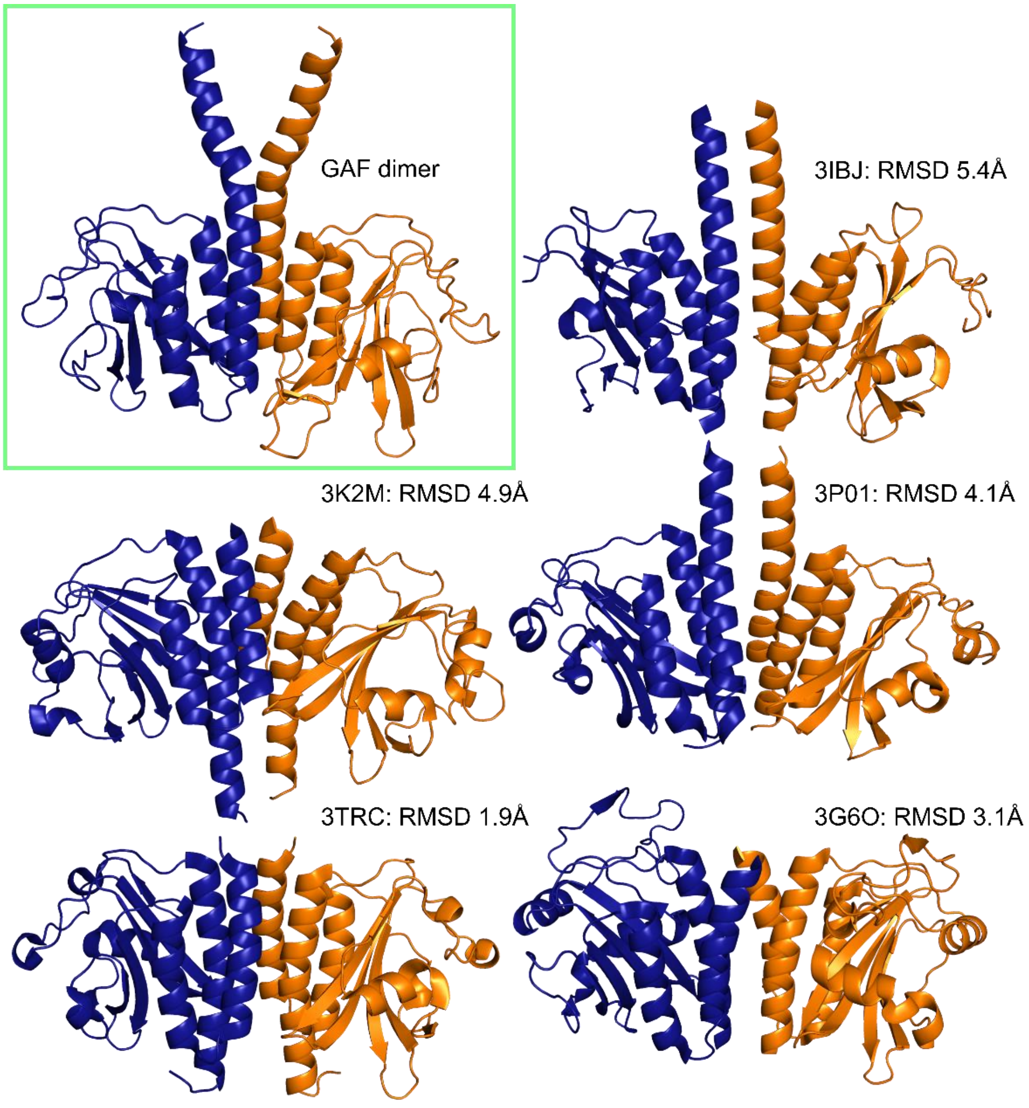

Supplementary Figure S6 | The homologous interfaces identified by TopDock and used to calculate protein–protein contacts for guided protein–protein docking. For each interface, the C_α RMSD to the GAF docking solution is shown. All five interfaces show similar folds and interaction patterns despite low sequence identity to the GAF domain.

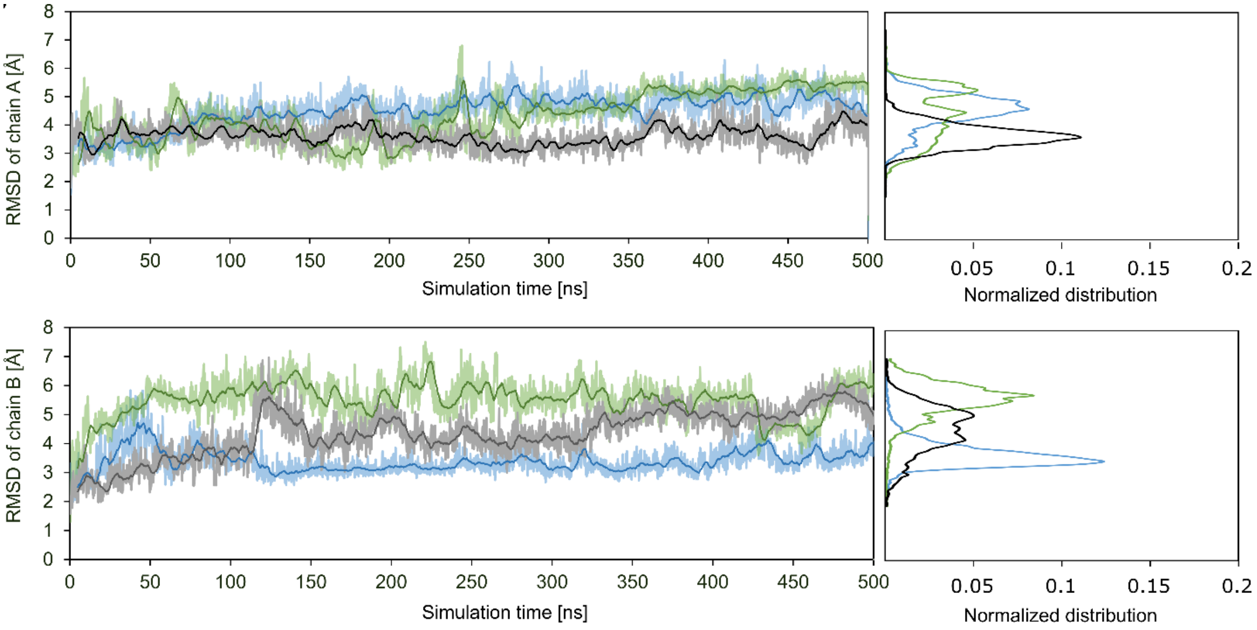

Supplementary Figure S7 | Root mean square deviations (RMSD) along three independent, unbiased MD simulations of the dimeric GAF domain of AtETR1 (amino acids 118 to 305) without ligand. RMSD values were calculated separately for chains A (top) and B (bottom) with respect to the starting structure, excluding unstructured loop regions (residues 222–290). The histograms to the right depict the frequency distributions of RMSD values within the single trajectories.

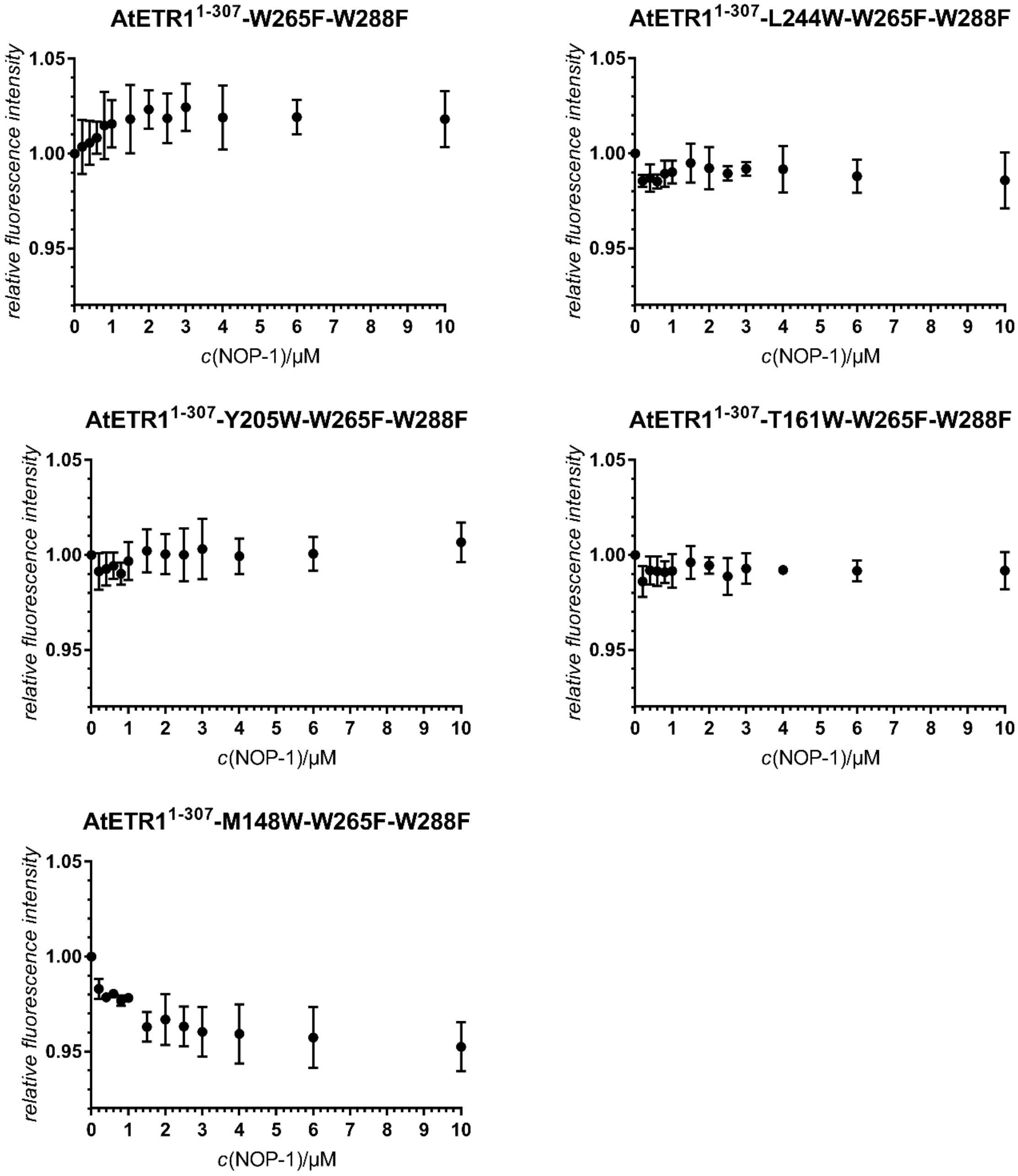

Supplementary Figure S8 | Binding of NOP-1 to AtETR1$^{1–307}$ Trp variants monitored by intrinsic tryptophan fluorescence. Excitation and emission wavelengths were 295 nm and 344 nm, respectively. Protein concentration was 1.0 µM.

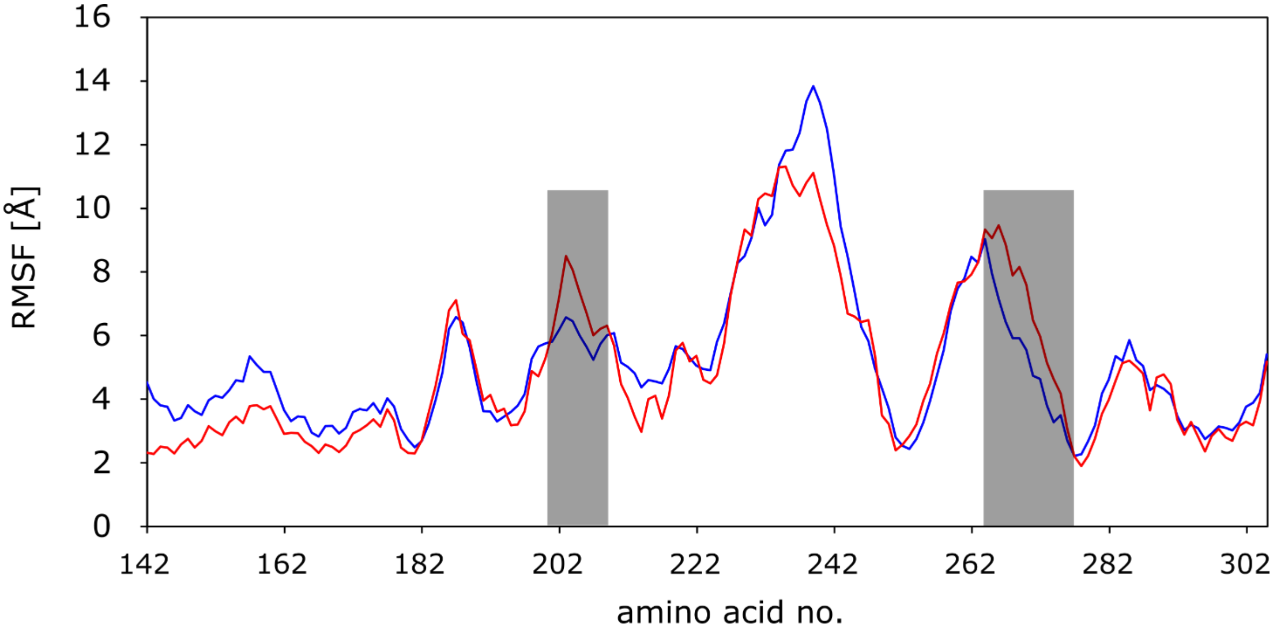

Supplementary Figure S9 | Root mean square fluctuations (RMSF) of the dimeric GAF domain of AtETR1 with (blue) and without (red) NOP-1 derived from MD simulations. The average RMSF value of chains A and B is depicted. Regions of residues with lower RMSF in the presence of NOP-1 are marked by grey boxes. These regions coincide with those identified by the rigidity and flexibility analysis (CNA; Fig. 4c,d in the main text) as having an increased structural stability upon NOP-1 binding.

Supplementary Table S2 | Molecular cloning scheme

Target plasmid	Template plasmid	Primer pair or *gene fragment**	
		**Fragment 1**	**Fragment 2**
pTEV-16b-AtETR1$^{1–589}$	pTEV-16b-AtETR1	ColE1-F<br>AtETR1-589-R	AtETR1-STOP-F<br>ColE1-R
pTEV-16b-AtETR1$^{1–407}$	pTEV-16b-AtETR1	ColE1-F<br>AtETR1-407-R	AtETR1-STOP-F<br>ColE1-R
pTEV-16b-AtETR1$^{1–307}$	pTEV-16b-AtETR1	ColE1-F<br>AtETR1-307-R	AtETR1-STOP-F<br>ColE1-R
pTEV-16b-AtETR1$^{1–157}$	pTEV-16b-AtETR1	ColE1-F<br>AtETR1-157-R	AtETR1-STOP-F<br>ColE1-R
pTEV-16b-AtETR1$^{306–738}$	pTEV-16b-AtETR1	ColE1-F<br>pET16b-TEV-R	AtETR1-306-F<br>ColE1-R
pTEV-16b-AtETR1$^{1–307}$-E177A-E178A-E246A-D283A	pTEV-16b-AtETR1$^{1–307}$	1_F_20170417<br>1_R_20170502	*A_20170417*<br>*(gene fragment)*
pTEV-16b-AtETR1$^{1–307}$-E152A-E169A	pTEV-16b-AtETR1$^{1–307}$	2_F_20170417<br>2_R_20170502	*B_20170417*<br>*(gene fragment)*
pTEV-16b-AtETR1$^{1–307}$-E190A-E204A	pTEV-16b-AtETR1$^{1–307}$	2_F_20170417<br>2_R_20170417	*C_20170417*<br>*(gene fragment*)
pTEV-16b-AtETR1$^{1–307}$-W265F-W288F	pTEV-16b-AtETR1$^{1–307}$	F_for_20170614<br>F_rev_20170614	*W265F-W288F*<br>*(gene fragment)*
pTEV-16b-AtETR1$^{1–307}$-L244W-W265F-W288F	pTEV-16b-AtETR1$^{1–307}$-W265F-W288F	ColE1-F<br>244_R	244_R<br>ColE1-R
pTEV-16b-AtETR1$^{1–307}$-Y205W-W265F-W288F	pTEV-16b-AtETR1$^{1–307}$-W265F-W288F	ColE1-F<br>205_R	205_F<br>ColE1-R
pTEV-16b-AtETR1$^{1–307}$-M148W-W265F-W288F	pTEV-16b-AtETR1$^{1–307}$-W265F-W288F	ColE1-F<br>148_F	148_R<br>ColE1-R
pTEV-16b-AtETR1$^{1–307}$-T161W-W265F-W288F	pTEV-16b-AtETR1$^{1–307}$-W265F-W288F	ColE1-F<br>161_R	161_F<br>ColE1-R

* Each target plasmid was assembled in a Gibson reaction from the two fragments. Fragments were either purchased as a synthetic double-stranded DNA (gBlocks gene fragments from Integrated DNA Technologies) or amplified in a PCR using an indicated pair of primers.

Supplementary Table S3 | Primers used in the molecular cloning

Primer	Sequence (5′ → 3′)
**ColE1-F**	GGAGCGAACGACCTACACCGAACTGAGATACCTACAGCG
**ColE1-R**	CGCTGTAGGTATCTCAGTTCGGTGTAGGTCGTTCGCTCC
**AtETR1-STOP-F**	TAAGGATCCGGCTGCTAACAAAGCCCGAAAG
**AtETR1-589-R**	TTGTTAGCAGCCGGATCCTTATTCGTTTGAACGTTCTGAGATCCCAAGTTTAAC
**AtETR1-407-R**	TTGTTAGCAGCCGGATCCTTAATCTTCTAACCTTGAAAGATCTAAGACATCATTCATC
**AtETR1-307-R**	TTGTTAGCAGCCGGATCCTTAGAGAGCTACAGCCACCTGATCAGC
**AtETR1-157-R**	TTGTTAGCAGCCGGATCCTTATAAAGTGCTTCTAATCTCATGAGTCAACATTCTCAC
**pET16b-TEV-R**	ATGTCCCTGAAAATACAGGTTTTCATGGCCGCTG
**AtETR1-306-F**	AACCTGTATTTTCAGGGACATGCTCTCTCACATGCTGCGATCCTAG
**1_F_20170417**	AGTGCAAGGCAATGGCATGTCCATGAG
**1_R_20170502**	CAAAGCTAATGTCCTACCAAGCTCAACAAG
**2_F_20170417**	TATACGGTTCCTATTCAATTACCGGTGATTAACC
**2_R_20170502**	ATGAGTCAACATTCTCACATGCCTTCCGGTTTC
**2_R_20170417**	GAAACCGGAAGGCATGTGAGAATGTTGACTCAT
**2_F_20170417**	TATACGGTTCCTATTCAATTACCGGTGATTAACC
**F_for_20170614**	GTCATTAATCTGAAAATTAGAAAGGTGGAGAAGCGGAAC
**F_rev_20170614**	TAAGGATCCGGCTGCTAACAAAGCCCGAAAG
**244_F**	ATGTGGGGGGAGGTGGTCGCTGTG
**244_R**	CCTCCCCCCACATATATTTCCCAGAAACAGG
**205_F**	CCCGTGGAGTGGACGGTTCCTATTCAATTAC
**205_R**	GGAACCGTCCACTCCACGGGATGTTG
**148_F**	GGCATGTGAGATGGTTGACTCATGAGATTAG
**148_R**	CCATCTCACATGCCTTCCGGTTTCTTCC
**161_F**	TGGATTTTAAAGACTACACTTGTTGAGCTTGGTAGGAC
**161_R**	GTCTTTAAAATCCAATGTCTATCTAAAGTGCTTCTAATCTCATG

Supplementary Table S4 | Gene fragments used in the molecular cloning

Gene fragment	Sequence (5′→3′)
**A_20170417**	CTTGGTAGGACATTAGCTTTGGCGGCGTGTGCATTGTGGATGCCTACTAGAACTGGGTTA<br>GAGCTACAGCTTTCTTATACACTTCGTCATCAACATCCCGTGGAGTATACGGTTCCTATTCA<br>ATTACCGGTGATTAACCAAGTGTTTGGTACTAGTAGGGCTGTAAAAATATCTCCTAATTCT<br>CCTGTGGCTAGGTTGAGACCTGTTTCTGGGAAATATATGCTAGGGGCGGTGGTCGCTGT<br>GAGGGTTCCGCTTCTCCACCTTTCTAATTTTCAGATTAATGACTGGCCTGAGCTTTCAACAA<br>AGAGATATGCTTTGATGGTTTTGATGCTTCCTTCAGCTAGTGCAAGGCAATGGCATGTC
**B_20170417**	CATGTGAGAATGTTGACTCATGCGATTAGAAGCACTTTAGATAGACATACTATTTTAAAG<br>ACTACACTTGTTGCGCTTGGTAGGACATTAGCTTTGGAGGAGTGTGCATTGTGGATGCCT<br>ACTAGAACTGGGTTAGAGCTACAGCTTTCTTATACACTTCGTCATCAACATCCCGTGGAGT<br>ATACGGTTCCTATTCAATTA
**C_20170417**	CATGTGAGAATGTTGACTCATGAGATTAGAAGCACTTTAGATAGACATACTATTTTAAAG<br>ACTACACTTGTTGAGCTTGGTAGGACATTAGCTTTGGAGGAGTGTGCATTGTGGATGCCT<br>ACTAGAACTGGGTTAGCGCTACAGCTTTCTTATACACTTCGTCATCAACATCCCGTGGCGT<br>ATACGGTTCCTATTCAATTA
**W265F-W288F**	TCTAATTTTCAGATTAATGACTTTCCTGAGCTTTCAACAAAGAGATATGCTTTGATGGTTTT<br>GATGCTTCCTTCAGATAGTGCAAGGCAATTCCATGTCCATGAGTTGGAACTCGTTGAAGT<br>CGTCGCTGATCAGGTGGCTGTAGCTCTCTAAGGATCCGGCTGCTAACAA

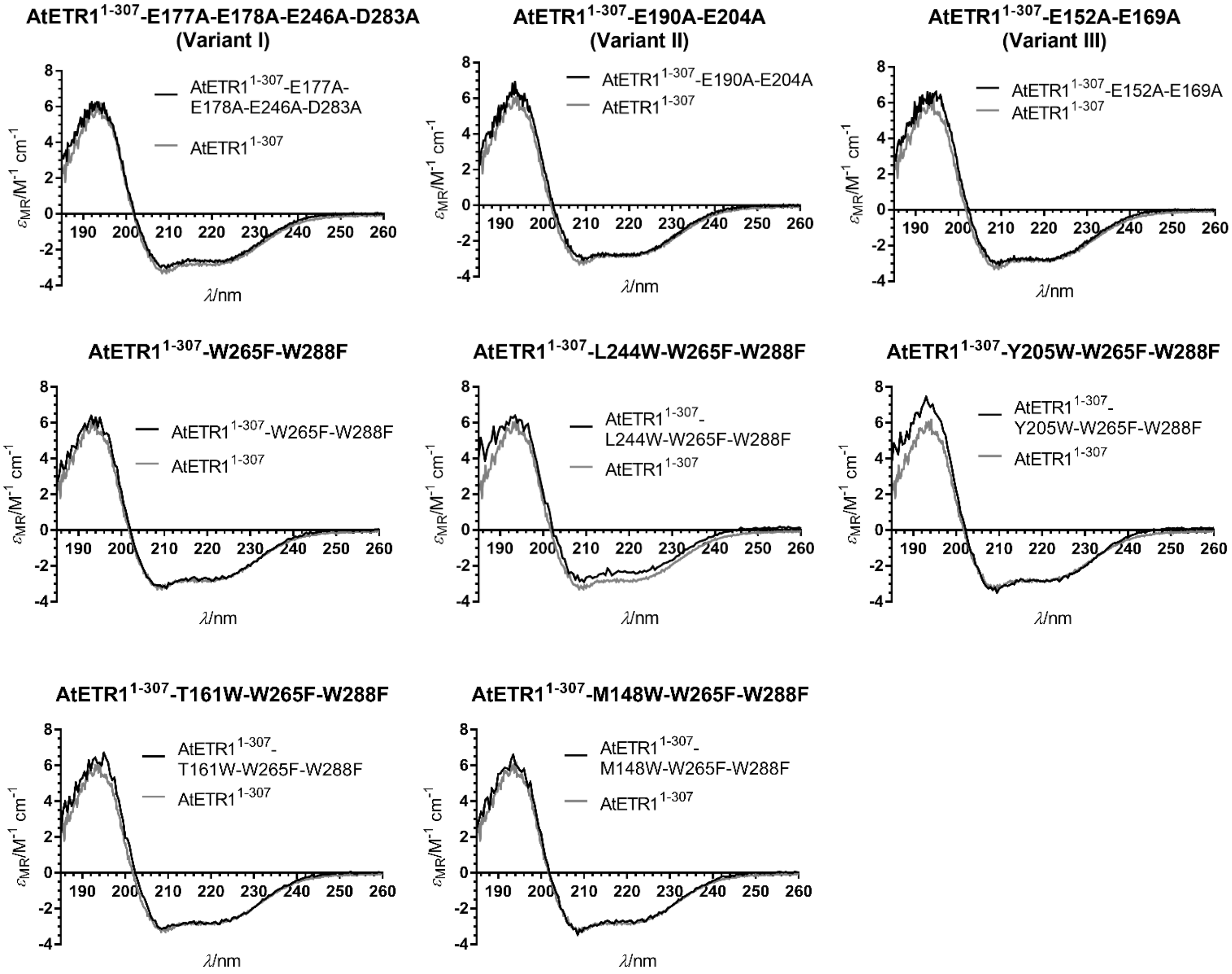

Supplementary Figure S10 | CD spectra of the AtETR1$^{1–307}$ variants. The spectrum of each variant (black curve) is compared with that of the unchanged AtETR1$^{1-307}$ (grey curve).

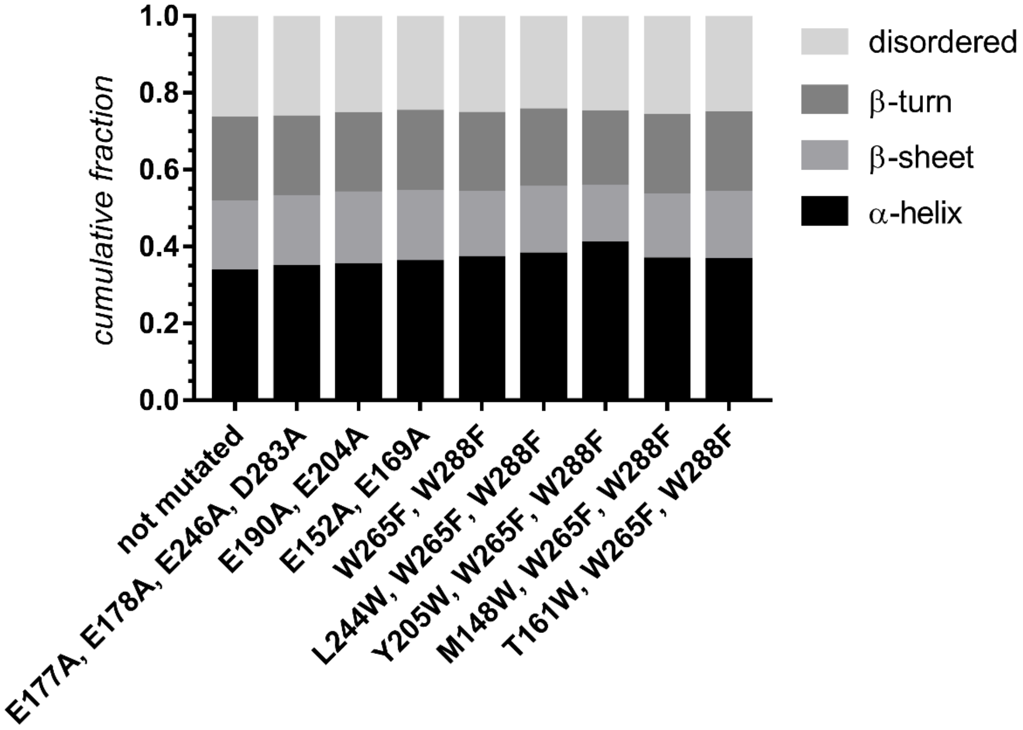

Supplementary Figure S11 | Secondary structure content of the not mutated AtETR1$^{1–307}$ and its variants. The plotted fractions are averaged values determined from the CD spectra by three different methods (as described in the Materials and Methods).

Supplementary References

1 Kabsch, W. & Sander, C. Dictionary of protein secondary structure: pattern recognition of hydrogen-bonded and geometrical features. *Biopolymers* **22**, 2577-2637, doi:10.1002/bip.360221211 (1983).

2 Perez-Iratxeta, C. & Andrade-Navarro, M. A. K2D2: estimation of protein secondary structure from circular dichroism spectra. *BMC Struct Biol* **8**, 25, doi:10.1186/1472-6807-8-25 (2008).

3 Boratyn, G. M. *et al.* Domain enhanced lookup time accelerated BLAST. *Biol. Direct* **7**, 12, doi:10.1186/1745-6150-7-12 (2012).

4 O'Sullivan, O., Suhre, K., Abergel, C., Higgins, D. G. & Notredame, C. 3DCoffee: combining protein sequences and structures within multiple sequence alignments. *J. Mol. Biol.* **340**, 385-395, doi:10.1016/j.jmp.2004.04.056 (2004).

5 Laskowski, R. A., MacArthur, M. W., Moss, D. S. & Thornton, J. M. PROCHECK: a program to check the stereochemical quality of protein structures. *J. Appl. Crystallogr.* **26**, 283-291, doi:Doi 10.1107/S0021889892009944 (1993).

6 Eddy, S. R. Accelerated profile HMM searches. *PLoS Comput. Biol.* **7**, e1002195, doi:10.1371/journal.pcbi.1002195 (2011).

7 Katoh, K. & Standley, D. M. MAFFT multiple sequence alignment software version 7: improvements in performance and usability. *Mol. Biol. Evol.* **30**, 772-780, doi:10.1093/molbev/mst010 (2013).

8 Chen, V. B. *et al.* MolProbity: all-atom structure validation for macromolecular crystallography. *Acta Crystallogr. Sect. D. Biol. Crystallogr.* **66**, 12-21, doi:10.1107/S0907444909042073 (2009).

9 Remmert, M., Biegert, A., Hauser, A. & Söding, J. HHblits: lightning-fast iterative protein sequence searching by HMM-HMM alignment. *Nat. Methods* **9**, 173-175, doi:10.1038/Nmeth.1818 (2012).

10 Collingridge, P. W. & Kelly, S. MergeAlign: improving multiple sequence alignment performance by dynamic reconstruction of consensus multiple sequence alignments. *BMC Bioinformatics* **13**, 117, doi:10.1186/1471-2105-13-117 (2012).

11 Melo, F. & Feytmans, E. Novel knowledge-based mean force potential at atomic level. *J. Mol. Biol.* **267**, 207-222, doi:DOI 10.1006/jmbi.1996.0868 (1997).

12 Söding, J., Biegert, A. & Lupas, A. N. The HHpred interactive server for protein homology detection and structure prediction. *Nucleic Acids Res.* **33**, W244-W248, doi:10.1093/nar/gki408 (2005).

13 Madhusudhan, M., Webb, B. M., Marti-Renom, M. A., Eswar, N. & Sali, A. Alignment of multiple protein structures based on sequence and structure features. *Protein Eng. Des. Sel.* **22**, 569-574, doi:10.1093/protein/gzp040 (2009).

14 Sippl, M. J. Recognition of errors in three-dimensional structures of proteins. *Proteins: Structure, Function, and Genetics* **17**, 355-362, doi:10.1002/prot.340170404 (1993).

15 Rychlewski, L., Li, W., Jaroszewski, L. & Godzik, A. Comparison of sequence profiles. Strategies for structural predictions using sequence information. *Protein Sci.* **9**, 232-241, doi:10.1110/ps.9.2.232 (2000).

16 Pei, J., Kim, B.-H. & Grishin, N. V. PROMALS3D: a tool for multiple protein sequence and structure alignments. *Nucleic Acids Res.* **36**, 2295-2300, doi:10.1093/nar/gkn072 (2008).

17 Shen, M. y. & Sali, A. Statistical potential for assessment and prediction of protein structures. *Protein Sci.* **15**, 2507-2524, doi:10.1110/ps.062416606 (2006).

18 Yang, Y., Faraggi, E., Zhao, H. & Zhou, Y. Improving protein fold recognition and template-based modeling by employing probabilistic-based matching between predicted one-dimensional

structural properties of query and corresponding native properties of templates. *Bioinformatics* **27**, 2076-2082, doi:10.1093/bioinformatics/btr350 (2011).
19 Daniels, N. M., Nadimpalli, S. & Cowen, L. J. Formatt: Correcting protein multiple structural alignments by incorporating sequence alignment. *BMC Bioinformatics* **13**, 259, doi:10.1186/1471-2105-13-259 (2012).
20 Zhou, H. & Zhou, Y. Distance-scaled, finite ideal-gas reference state improves structure-derived potentials of mean force for structure selection and stability prediction. *Protein Sci.* **11**, 2714-2726, doi:10.1110/ps.0217002 (2002).
21 Peng, J. & Xu, J. RaptorX: exploiting structure information for protein alignment by statistical inference. *Proteins: Structure, Function, and Bioinformatics* **79**, 161-171, doi:10.1002/prot.23175 (2011).
22 Konagurthu, A. S., Whisstock, J. C., Stuckey, P. J. & Lesk, A. M. MUSTANG: a multiple structural alignment algorithm. *Proteins: Structure, Function, and Bioinformatics* **64**, 559-574, doi:10.1002/prot.20921 (2006).
23 McGuffin, L. J. & Roche, D. B. Rapid model quality assessment for protein structure predictions using the comparison of multiple models without structural alignments. *Bioinformatics* **26**, 182-188, doi:10.1093/bioinformatics/btp629 (2010).
24 Wu, S. & Zhang, Y. LOMETS: a local meta-threading-server for protein structure prediction. *Nucleic Acids Res.* **35**, 3375-3382, doi:10.1093/nar/gkm251 (2007).
25 Wang, S., Peng, J. & Xu, J. Alignment of distantly related protein structures: algorithm, bound and implications to homology modeling. *Bioinformatics* **27**, 2537-2545, doi:10.1093/bioinformatics/btr432 (2011).
26 Wallner, B. & Elofsson, A. Identification of correct regions in protein models using structural, alignment, and consensus information. *Protein Sci.* **15**, 900-913, doi:10.1110/ps.051799606 (2006).
27 Lobley, A., Sadowski, M. I. & Jones, D. T. pGenTHREADER and pDomTHREADER: new methods for improved protein fold recognition and superfamily discrimination. *Bioinformatics* **25**, 1761-1767, doi:10.1093/bioinformatics/btp302 (2009).
28 Zhang, Y. & Skolnick, J. SPICKER: A clustering approach to identify near-native protein folds. *J. Comput. Chem.* **25**, 865-871, doi:10.1002/jcc.20011 (2004).
29 Benkert, P., Schwede, T. & Tosatto, S. C. QMEANclust: estimation of protein model quality by combining a composite scoring function with structural density information. *BMC Struct. Biol.* **9**, 35, doi:10.1186/1472-6807-9-35 (2009).
30 Pearson, W. R. Finding protein and nucleotide similarities with FASTA. *Current protocols in bioinformatics* **Chapter 3**, 3.9. 1-3.9. 25, doi:10.1002/0471250953.bi0309s04 (2004).
31 Ray, A., Lindahl, E. & Wallner, B. Improved model quality assessment using ProQ2. *BMC Bioinformatics* **13**, 224, doi:10.1186/1471-2105-13-224 (2012).
32 Karplus, K. SAM-T08, HMM-based protein structure prediction. *Nucleic Acids Res.* **37**, gkp403, doi:10.1093/nar/gkp403 (2009).
33 Randall, A. & Baldi, P. SELECTpro: effective protein model selection using a structure-based energy function resistant to BLUNDERs. *BMC Struct. Biol.* **8**, 52, doi:10.1186/1472-6807-8-52 (2008).
34 Thompson, J. D., Higgins, D. G. & Gibson, T. J. CLUSTAL W: improving the sensitivity of progressive multiple sequence alignment through sequence weighting, position-specific gap penalties and weight matrix choice. *Nucleic Acids Res.* **22**, 4673-4680, doi:10.1093/nar/22.22.4673 (1994).
35 Lee, C., Grasso, C. & Sharlow, M. F. Multiple sequence alignment using partial order graphs. *Bioinformatics* **18**, 452-464, doi:10.1093/bioinformatics/18.3.452 (2002).

36 Edgar, R. C. MUSCLE: multiple sequence alignment with high accuracy and high throughput. *Nucleic Acids Res.* **32**, 1792-1797, doi:10.1093/nar/gkh340 (2004).

37 Sierk, M. L., Smoot, M. E., Bass, E. J. & Pearson, W. R. Improving pairwise sequence alignment accuracy using near-optimal protein sequence alignments. *BMC Bioinformatics* **11**, 146, doi:10.1186/1471-2105-11-146 (2010).

38 Pei, J., Sadreyev, R. & Grishin, N. V. PCMA: fast and accurate multiple sequence alignment based on profile consistency. *Bioinformatics* **19**, 427-428, doi:10.1093/bioinformatics/btg008 (2003).

39 Do, C. B., Mahabhashyam, M. S., Brudno, M. & Batzoglou, S. ProbCons: Probabilistic consistency-based multiple sequence alignment. *Genome Res.* **15**, 330-340, doi:10.1101/gr.2821705 (2005).

40 Al Ait, L., Yamak, Z. & Morgenstern, B. DIALIGN at GOBICS—multiple sequence alignment using various sources of external information. *Nucleic Acids Res.* **41**, W3-W7, doi:10.1093/nar/gkt283 (2013).

41 Taylor, W. R. Protein structure comparison using iterated double dynamic programming. *Protein Sci.* **8**, 654-665, doi:10.1110/ps.8.3.654 (1999).

42 Zhang, Y. & Skolnick, J. TM-align: a protein structure alignment algorithm based on the TM-score. *Nucleic Acids Res.* **33**, 2302-2309, doi:10.1093/nar/gki524 (2005).
